# Auxin response and PIN‐mediated transport in chlorophyte algae

**DOI:** 10.1111/jipb.70309

**Published:** 2026-06-10

**Authors:** Adrijana Smoljan, Sarah Koutnik‐Abele, Dmitrii Vladimirtsev, Petr Klíma, Anita Bírošíková, Yuzhou Zhang, Jack Merrin, Maximilian Schuster, Katarina Kurtović, Ulrich Z. Hammes, Jan Petrášek, Jiří Friml

**Affiliations:** ^1^ Institute of Science and Technology Austria (ISTA) Klosterneuburg 3400 Austria; ^2^ School of Life Sciences Weihenstephan, Plant Systems Biology Technical University of Munich Freising 85354 Germany; ^3^ Institute of Experimental Botany Czech Academy of Sciences Prague 165 00 Czech Republic; ^4^ State Key Laboratory for Crop Stress Resistance and High‐Efficiency Production, College of Life Sciences Northwest A&F University Yangling 712100 China; ^5^ Institute of Future Agriculture Northwest A&F University Yangling 712100 China; ^6^ Department of Experimental Plant Biology, Faculty of Science Charles University Prague 128 44 Czech Republic

**Keywords:** auxin transport, chlorophyte algae, indole‐3‐acetic acid, PIN proteins, plant evolution, polar auxin transport

## Abstract

Auxin, primarily indole‐3‐acetic acid (IAA), is a central regulator of growth and development in land plants, but its physiological role in chlorophyte algae remains unclear. Here, we show that exogenous IAA modulates growth in *Chlorella sorokiniana*, *Chlorella variabilis*, and *Chlamydomonas reinhardtii* in a concentration‐dependent manner. Low IAA concentrations promoted growth by accelerating the onset of cell division without affecting cell size, whereas higher concentrations inhibited proliferation. Radiotracer assays showed that all three species take up and release IAA across the plasma membrane through a combination of passive diffusion and energy‐dependent, saturable processes. Competition by excess unlabeled natural and synthetic auxins further supported the presence of carrier‐mediated transport with broad substrate recognition. Phylogenetic analyses identified potential PIN‐like auxin exporters in chlorophytes and other non‐plant eukaryotes, and structural modeling supported conservation of the overall PIN fold and predicted auxin‐binding residues. However, functional assays in *Xenopus laevis* oocytes, tobacco BY‐2 cultured cells, and *Arabidopsis thaliana* did not support a role for these proteins in directional auxin export. Instead, non‐plant PIN homologs localized predominantly to the endoplasmic reticulum and showed limited or no transport activity in heterologous systems. Together, these findings indicate that auxin responsiveness and basic cellular auxin transport predate canonical PIN‐mediated directional auxin export, which appears to be a later innovation of the streptophyte lineage.

## Graphical abstract

Like multicellular plants, green algae respond to auxin and move it across their cells. However, their PIN‐like proteins do not act like plant auxin exporters, suggesting that basic auxin transport evolved early and specialized directional transport appeared later in plant evolution.

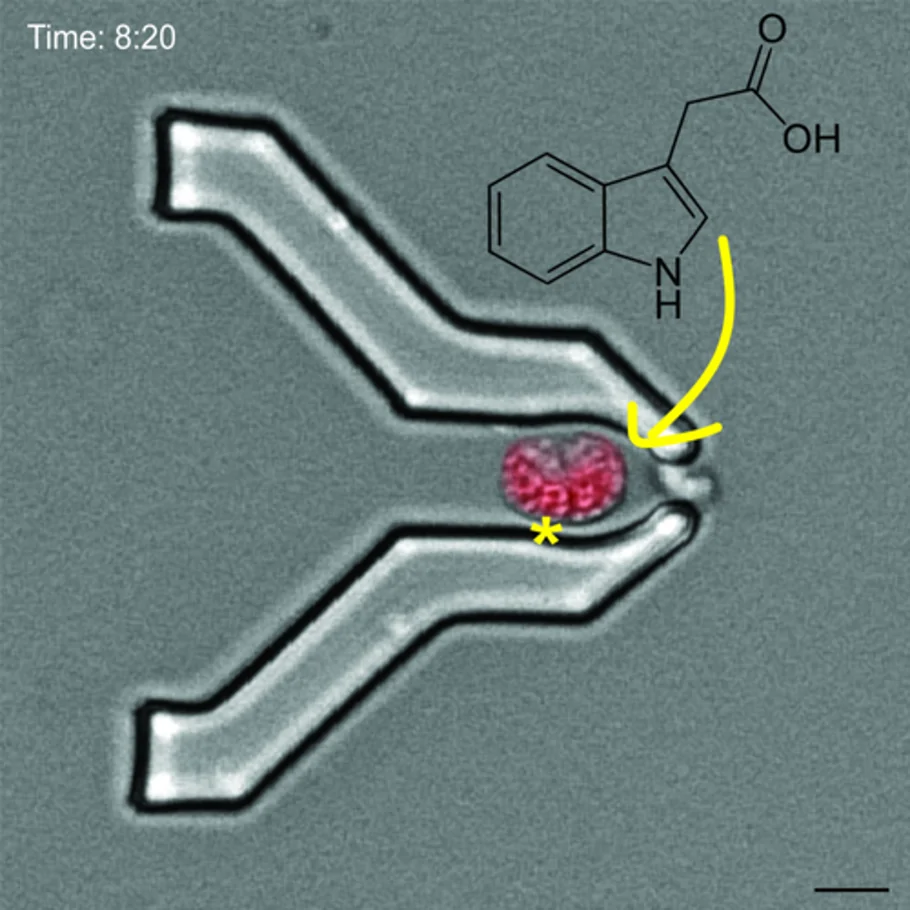

## INTRODUCTION

The transition of plants from aquatic to terrestrial environments, which occurred in a streptophyte algae, was accompanied by a series of developmental innovations that underpinned the establishment of complex plant body plans and adaptive growth strategies (Harrison, 2017; Donoghue et al., 2021; Bowman, 2022). Central to these innovations is the phytohormone auxin, which orchestrates plant development through local biosynthesis and tightly regulated directional transport. These activities result in local auxin maxima and concentration gradients essential for morphogenesis and environmental responses (Friml, 2022).

Indole‐3‐acetic acid (IAA), the main form of auxin in land plants, has been detected in diverse green algal lineages, including chlorophytes such as *Chlorella vulgaris*, *Scenedesmus obliquus*, and *Chlamydomonas reinhardtii* (Basu et al., 2002; Le Bail et al., 2010; Bogaert et al., 2019). A recent broad phytohormone‐profiling study across Viridiplantae found free IAA in numerous charophyte and chlorophyte taxa, arguing that IAA biosynthesis and presence preceded land‐plant evolution (Skokan et al., 2019). Beyond the plant kingdom, IAA and auxin‐related compounds are found across multiple domains of life, including bacteria, archaea, fungi, and algae (Cohen et al., 2002; Chanclud and Morel, 2016; Bogaert et al., 2019; Zhang et al., 2022). In prokaryotes, IAA biosynthesis is widespread and often linked to interactions with plants, whether as pathogens, symbionts, or plant‐growth‐promoting rhizobacteria (Amin et al., 2015; Cox et al., 2018). Therefore, the detection of IAA in algal cultures must be interpreted cautiously, as both bacteria and fungi can produce IAA independently (Chanclud and Morel, 2016; Zhang et al., 2022), making contamination a persistent concern.

Physiological responses of green algae to exogenous IAA tentatively indicate IAA perception and signaling capacity (Bagwell et al., 2014; Piotrowska‐Niczyporuk and Bajguz, 2014; Calatrava et al., 2024). IAA application was shown to promote cell enlargement in *Chlorella vulgaris*, with higher concentrations inhibiting growth, paralleling similar effects in higher plants (Piotrowska‐Niczyporuk and Bajguz, 2014). Recent studies report that *Chlorella sorokiniana* accumulates and releases IAA during active growth (Khasin et al., 2018), and that green algae exhibit dose‐dependent responses to auxin, including increased biomass and storage compounds at low doses and growth inhibition at high doses (Piotrowska‐Niczyporuk and Bajguz, 2014).

In land plants, auxin's developmental functions are tightly linked with directional, intercellular transport, primarily mediated by the PIN family of auxin transporters (Luschnig and Friml, 2024). PIN proteins play central roles in organogenesis, tissue patterning, and tropic responses by enabling the formation of directional auxin gradients. Recent studies have elucidated the structural basis of PIN‐mediated transport and uncovered regulatory mechanisms that control their activity and subcellular localization (Su et al., 2022; Ung et al., 2022; Yang et al., 2022). Evidence from the filamentous streptophyte alga *Klebsormidium flaccidum* suggests that functional PIN‐mediated transport predates the evolution of land plants, as demonstrated by the ability of KfPIN to mediate auxin efflux in multiple heterologous expression systems, including plant and animal cells (Skokan et al., 2019). However, the evolutionary origin of PIN‐mediated auxin transport remains unresolved, since PIN homologs are also found in earlier‐diverging lineages, making it unclear when and how directional auxin transport first evolved.

Besides streptophyte green algae, more distant potential PIN homologs were also identified in several chlorophytes, including *C. sorokiniana* and *Chlorella variabilis*, using Arabidopsis PIN sequences as queries. In contrast, *C. reinhardtii* and other chlorophyte genomes yielded only highly diverged sequences that failed reciprocal BLAST validation, suggesting that clear PIN orthologs are restricted to *Chlorella* species, whereas other chlorophyte sequences likely represent more distantly related or functionally distinct transporters (Vosolsobě et al., 2020).

In this study, we investigate how chlorophyte algae respond to auxin and whether PIN‐like proteins from chlorophytes and non‐plant lineages function as auxin transporters. By combining physiological assays, radiotracer‐based transport measurements, phylogenetic and structural analyses, and heterologous expression systems, we test whether PIN‐like proteins outside the streptophyte lineage contribute to directional auxin export. Our results define the capacity of chlorophytes to respond to and transport auxin while clarifying the functional limits of non‐plant PIN‐like proteins, thereby clarifying which aspects of PIN‐mediated auxin transport are conserved and which are specific to the plant lineage.

## RESULTS

### Auxin modulates chlorophyte cell proliferation in a concentration‐dependent manner

To investigate whether chlorophyte algae respond to external IAA, we treated cultures of *C. sorokiniana*, *C. variabilis*, and *C. reinhardtii* with a range of IAA concentrations. Cell density, measured as cells per mL using an automated cell counter (Figure 1A–C), revealed a concentration‐dependent growth response. At 1 μM IAA, all three species showed increased cell numbers compared with the untreated control (mock), whereas 50 μM IAA significantly reduced cell proliferation. Notably, 10 μM IAA treatment did not differ from mock, suggesting a transition between stimulatory and inhibitory concentration ranges between 10 and 50 μM IAA. Tryptophan treatment, included as a precursor control, did not influence growth compared with mock (Figure S1A–C), consistent with a direct response to auxin.

**Figure 1:**
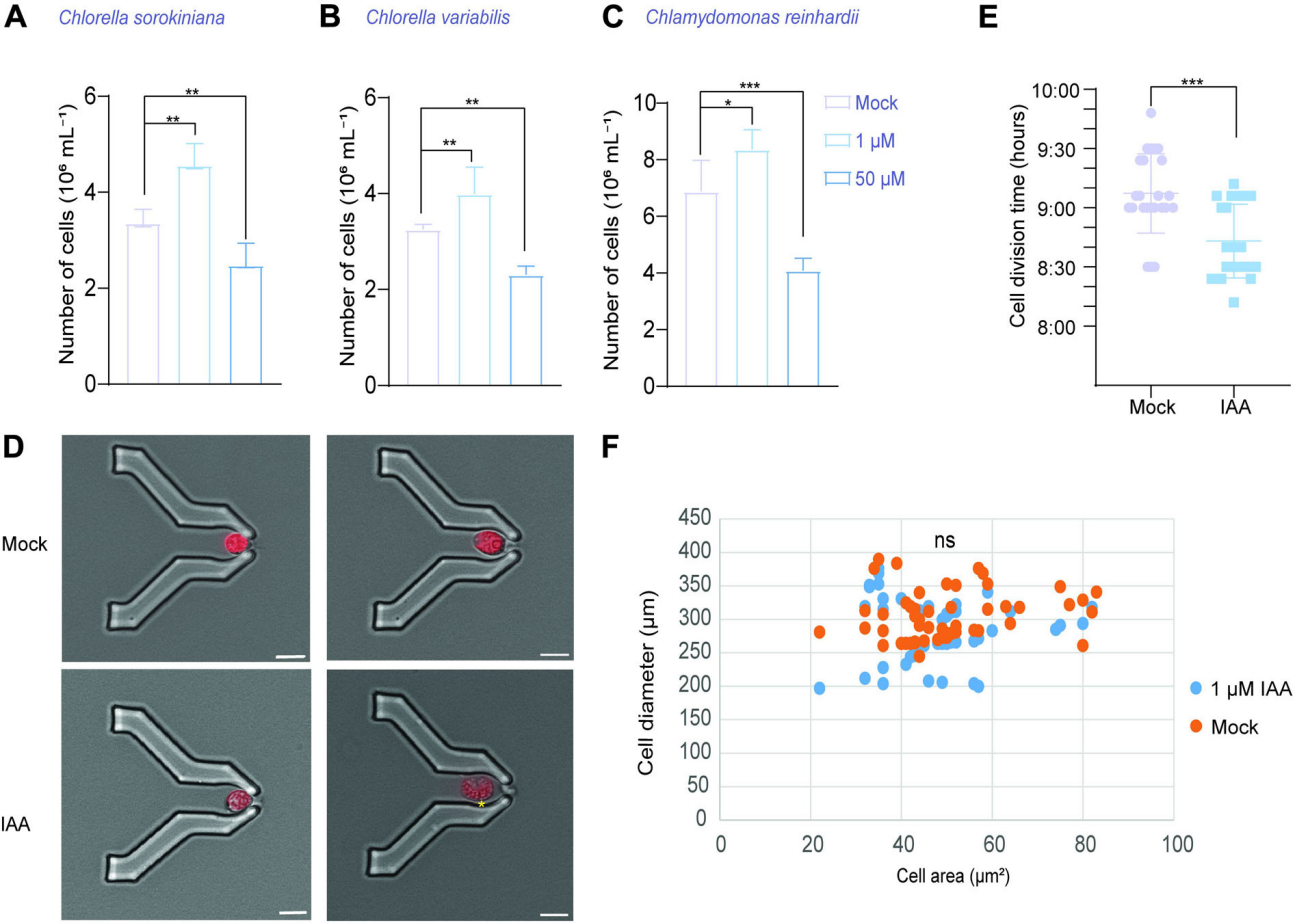
Effects of auxin on cell number, division timing, and cell size in green algae **(A**–**C)** Cell numbers on Day 2 for *Chlorella sorokiniana* **(A)**, *Chlorella variabilis* **(B)**, and *Chlamydomonas reinhardtii* **(C)** treated with mock, 1 μM IAA, or 50 μM IAA. For *C. sorokiniana*, one‐way ANOVA showed a significant treatment effect (*F*(2, 12) = 33.29, *P* < 0.0001); Dunnett's test indicated increased cell number at 1 μM IAA (*P* = 0.0010) and decreased number at 50 μM (*P* = 0.0091). For *C. variabilis*, treatment also had a significant effect (*F*(2, 12) = 30.31, *P* < 0.0001); Dunnett's test revealed a similar pattern with increased cell number at 1 μM IAA (*P* = 0.0095) and reduction at 50 μM (*P* = 0.0018). Equal variance was confirmed for *C. sorokiniana*, but Bartlett's test indicated unequal variance for *C. variabilis*. **(D)** Representative images of *C. reinhardtii* cells in Tulip microfluidic chambers under mock or 1 μM IAA. The image shown corresponds to 8 h 40 min after treatment. Scale bar = 10 μm. **(E)** Time of first cell division was significantly earlier under IAA treatment (median 8:40 h) compared to mock (median 9:10 h; *P* = 0.0015, Mann–Whitney test, *n* = 30). **(F)** Single‐cell size distribution: cell area (μm^2^) vs. diameter (μm). Orange dots = mock; blue dots = 1 μM IAA. No significant difference between treated and wild‐type cells (Mann–Whitney test, *P* = 0.312).

Because OD_750_ and cell number do not always correlate, we also measured optical density as an independent estimate of biomass (Figure S1D–F). OD_750_ showed the same overall trend as cell counts: 1 μM IAA increased biomass, 50 μM decreased it, and 10 μM showed no significant effect. The intermediate concentration of 10 μM may therefore represent a transition point at which stimulatory and inhibitory effects balance each other, resulting in no measurable change in biomass compared with untreated controls. Together, these results confirm that auxin influences algal growth in a concentration‐dependent manner, consistent with previous reports in chlorophytes (Bagwell et al., 2014; Piotrowska‐Niczyporuk and Bajguz, 2014).

Having assessed culture growth, we next examined whether IAA affects the timing of cell division at the single‐cell level. We established live‐cell imaging using a custom‐designed Tulip microfluidic device, enabling long‐term observation under controlled treatment conditions (Figure 1D; Videos S1, S2). In *C. reinhardtii*, cells treated with 1 μM IAA initiated their first division significantly earlier than mock‐treated controls. Division onset was defined as the first visible division event observed by time‐lapse microscopy. This effect was quantified in Figure 1E, which shows the timing of first division across individual cells and reveals a significant shift toward earlier division onset under auxin treatment. Despite the earlier initiation of division, cell size at division was comparable between mock‐ and IAA‐treated cells (Figure 1F), indicating that auxin affects division timing rather than cell size increase. In contrast, higher IAA concentrations inhibited overall culture growth.

To determine whether high auxin concentrations directly affect cell division at the single‐cell level, we performed live‐cell imaging in Tulip‐shaped microfluidic chambers using 50 μM IAA (Figure S2). Under mock‐treated conditions, all cells underwent division within a defined time window between 510 and 570 min (8.5–9.5 h) after the start of imaging. In contrast, cells treated with 50 μM IAA did not exhibit any division events during the entire imaging period of 630 min (10.5 h). Notably, cells progressively lost fluorescence signal over time, precluding reliable tracking beyond this time point. These results demonstrate that high auxin concentrations strongly inhibit cell division under the tested conditions.

Together, these data indicate that low concentrations of IAA promote chlorophyte algal growth by advancing the onset of cell division without altering cell size, whereas higher concentrations inhibit proliferation.

### Active and passive auxin import and export in Chlamydomonas and Chlorella

To determine whether chlorophyte algae are capable of auxin transport, we performed uptake and export assays with radiolabeled IAA in *C. reinhardtii*, *Chlorella sorokiniana*, and *C. variabilis*. For uptake measurements, cells were incubated with [^3^H]‐IAA for 20 min, and intracellular radioactivity was quantified after washing to remove unbound IAA (Figure 2A). To assess export, cells were first loaded with [^3^H]‐IAA for 20 min, then washed and transferred to fresh medium for an additional 20 min before measuring the radioactivity released into the medium (Figure 2B). Uptake and export values were quantified relative to DMSO‐treated control cells, which served as the reference condition for normal transport activity. All three species accumulated and released radiolabeled IAA, indicating that IAA can cross the plasma membrane (PM). However, these assays alone could not distinguish between passive diffusion and carrier‐mediated transport.

**Figure 2:**
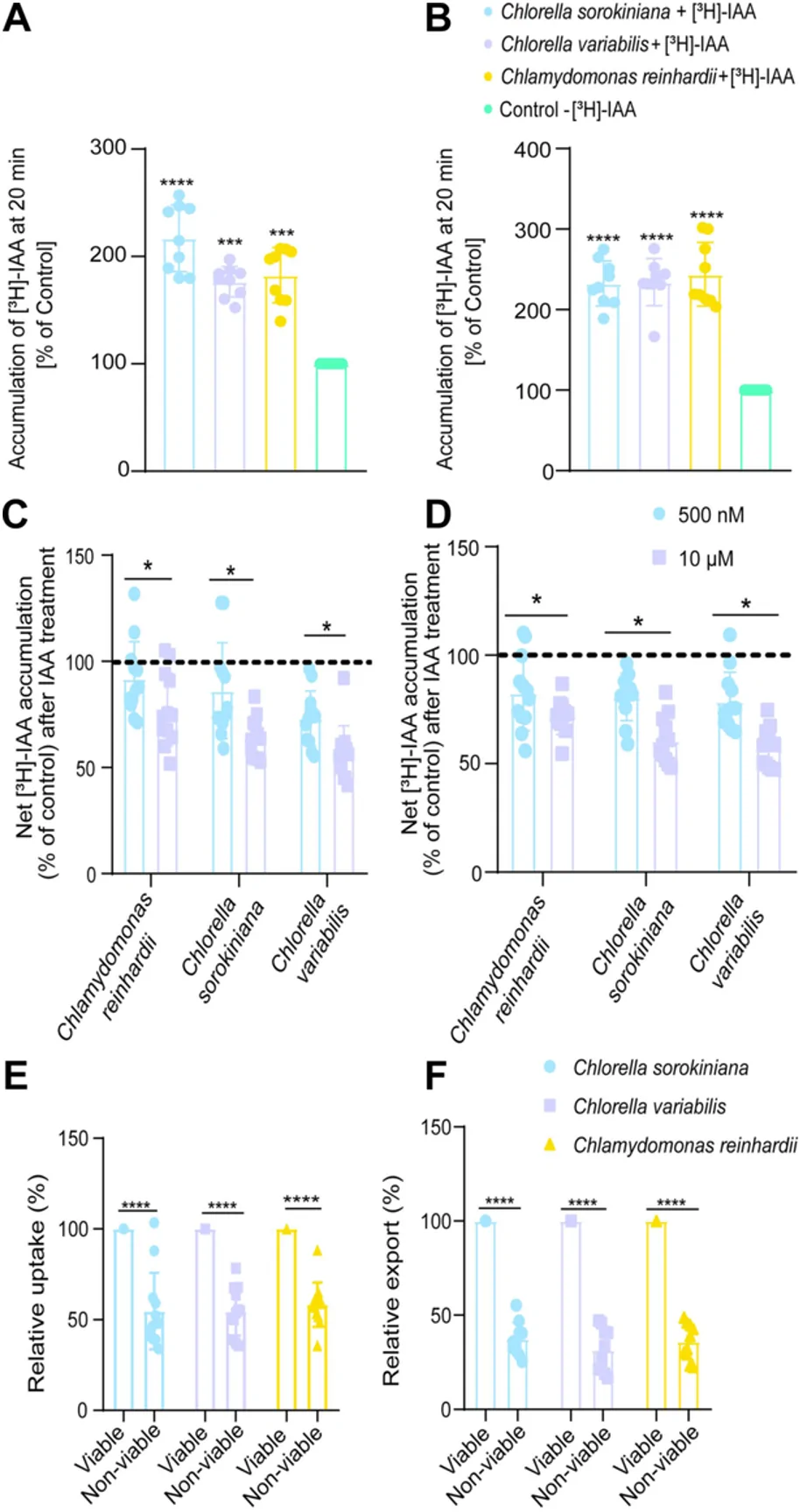
Auxin uptake and export in green algae after 20 min of incubation **(A)** Uptake of radiolabeled indole‐3‐acetic acid ([^3^H]‐IAA) after 20 min of incubation in *Chlamydomonas reinhardtii*, *Chlorella sorokiniana*, and *Chlorella variabilis*. Intracellular radioactivity is shown relative to DMSO‐treated vehicle controls, which were set to 100%. One‐way ANOVA revealed a significant treatment effect (*F*(3, 32) = 47.85, *P* < 0.0001, *R* ^2^ = 0.82). Dunnett's multiple comparisons test indicated significantly increased uptake in all three species compared with the control (****P < 0.0001). Variances were unequal (Brown–Forsythe test, *P* = 0.0005). **(B)** Export of radiolabeled IAA into the extracellular medium after 20 min, expressed relative to DMSO‐treated vehicle controls (set to 100%). One‐way ANOVA showed significant differences between treatments (*F*(3, 32) = 53.08, *P* < 0.0001, *R* ^2^ = 0.83). Dunnett's test confirmed significantly increased export in all species compared with the control (****P < 0.0001). Variances were unequal (Brown–Forsythe test, *P* = 0.0352). **(C)** Uptake competition assay performed in the presence of excess unlabeled IAA (500 nM or 10 μM) together with 5 nM [^3^H]‐IAA. Uptake is expressed as a percentage of the corresponding DMSO‐treated control. A paired *t*‐test showed significantly reduced uptake at 10 μM compared with 500 nM (*t*(2) = 7.506, *P* = 0.0173, *R* ^2^ = 0.97), with a mean difference of −17.81%. **(D)** Export competition assay under the same conditions as in **(C)**. Export into the medium was significantly reduced at 10 μM compared with 500 nM unlabeled IAA (*t*(2) = 5.121, *P* = 0.0361, *R* ^2^ = 0.93), with a mean difference of −17.54%. **(E)** Auxin uptake after 20 min in viable (untreated) versus sodium azide‐treated (ATP‐depleted) cells. Uptake in viable cells was normalized to 100%, and uptake in sodium azide‐treated cells is shown as relative influx. A significant treatment effect was detected (two‐way ANOVA, *P* < 0.0001), with no significant interaction or species effect. **(F)** Auxin export after 20 min in viable versus sodium azide‐treated cells. Export in viable cells was normalized to 100%, and export in sodium azide‐treated cells is shown as relative efflux. A significant treatment effect was detected (two‐way ANOVA, *P* < 0.0001), with no significant interaction or species effect.

To determine whether the selected incubation time was sufficient to achieve near‐saturating intracellular IAA levels, we performed a time‐course analysis of [^3^H]‐IAA accumulation in *C. reinhardtii* cells at 0, 2, 5, 10, 15, and 20 min (Figure S3). IAA uptake increased rapidly during the first 10 min and subsequently approached a plateau, with no significant differences observed between the 10, 15, and 20 min time points. These results indicate that a 10 min feeding period is sufficient to reach near‐saturating intracellular IAA levels under our experimental conditions, minimizing the possibility that differences in efflux arise from variation in initial uptake.

To test whether uptake and export involve specific transporters, we conducted competition assays using excess unlabeled IAA (cold IAA) at two concentrations (500 nM and 10 μM) in the presence of 5 nM [^3^H]‐IAA (Figure 2C, D). Pretreatment with cold IAA significantly reduced both radiolabeled auxin uptake and export into the medium, with a stronger effect observed at the higher concentration. This competitive inhibition is consistent with the presence of saturable transport components, as expected for carrier‐mediated auxin transport. In this process, transporters become occupied by unlabeled IAA, thereby reducing radiolabeled IAA flux.

To further assess substrate specificity of the transport system, we performed competition assays using the synthetic auxin 1‐naphthaleneacetic acid (NAA) (Figure S4). Pretreatment with NAA resulted in a comparable reduction in [^3^H]‐IAA uptake and export to that observed with excess unlabeled IAA. This finding supports the presence of a carrier‐mediated transport component and indicates that the underlying transport system is not strictly specific for IAA, but can recognize structurally related auxin analogs. Such substrate flexibility is consistent with studies in land plants, where auxin transport systems accommodate both natural and synthetic auxins with overlapping substrate specificity (Fukui et al., 2018).

To further investigate the energy dependency of auxin transport, cells were pretreated with sodium azide. Sodium azide is a well‐established metabolic inhibitor that blocks respiration and ATP synthesis by inhibiting cytochrome *c* oxidase and other energy‐dependent enzymes, while also affecting reactive oxygen species‐scavenging pathways (Nicholls, 1964; Maison‐Peteri and Etienne, 1977; Fei et al., 2000; Bowler et al., 2006). Microscopy of azide‐treated cells in Tulip microfluidic devices revealed disrupted chloroplast integrity, with chlorophyll autofluorescence condensed into punctate structures, while overall cell shape and cell wall integrity were preserved (Figure S2A). This indicates that although cellular energy metabolism was compromised, PM integrity remained intact, allowing meaningful interpretation of transport measurements.

To further assess the physiological state of azide‐treated cells, we performed fluorescein diacetate (FDA) staining and quantified the proportion of fluorescent cells (Figure S5). In untreated controls, 52 out of 60 cells (86.7%) were FDA‐positive, whereas after sodium azide pretreatment, only 22 out of 60 cells (36.7%) remained fluorescent. This statistically significant reduction indicates strong suppression of intracellular metabolic activity under ATP‐depleting conditions. Importantly, despite this reduction in FDA fluorescence, cells retained their overall morphology during the assay period, arguing against immediate gross cellular disintegration.

In all three species, radiolabeled IAA uptake was significantly reduced following azide treatment, reaching approximately 50% of DMSO‐treated control levels (Figure 2E). Export into the medium was even more strongly impaired (Figure 2F), consistent with the higher energy requirement for auxin export relative to uptake. Notably, residual IAA uptake persisted under ATP‐depleted conditions, indicating that passive diffusion contributes to overall auxin flux in chlorophyte algae.

Together, these results indicate that *Chlamydomonas* and *Chlorella* species transport IAA across the plasma membrane through a combination of passive diffusion and carrier‐mediated, energy‐dependent processes. Competitive inhibition by excess unlabeled IAA and NAA supports the presence of saturable transport components with broader substrate recognition, while time‐course analysis confirms that intracellular IAA levels reach near‐saturation under the applied experimental conditions. Reduced uptake and export following ATP depletion, together with suppressed metabolic activity in azide‐treated cells, further support an energy‐dependent contribution to auxin transport. Collectively, these findings provide strong evidence for auxin import and export systems in chlorophyte algae.

### Phylogenetic relationships, expression, and gene structure of PIN homologs

As PIN auxin exporters are key components of auxin transport in streptophytes, we investigated the evolutionary distribution and diversity of PIN homologs across eukaryotes. We performed a comprehensive phylogenetic analysis across 21 species spanning angiosperms, early‐diverging land plants, green algae, fungi, and protists (Figure 3). Representative PIN homologs were identified using an HMMER‐based search seeded with *Arabidopsis thaliana* PIN sequences. To reduce redundancy, sequences were clustered using USEARCH at 45% identity, yielding approximately 70 representative sequences for phylogenetic reconstruction. Short PINs (PIN5, PIN6, and PIN8), which differ in length and domain architecture from canonical PIN exporters, were excluded from the main phylogenetic analysis.

**Figure 3:**
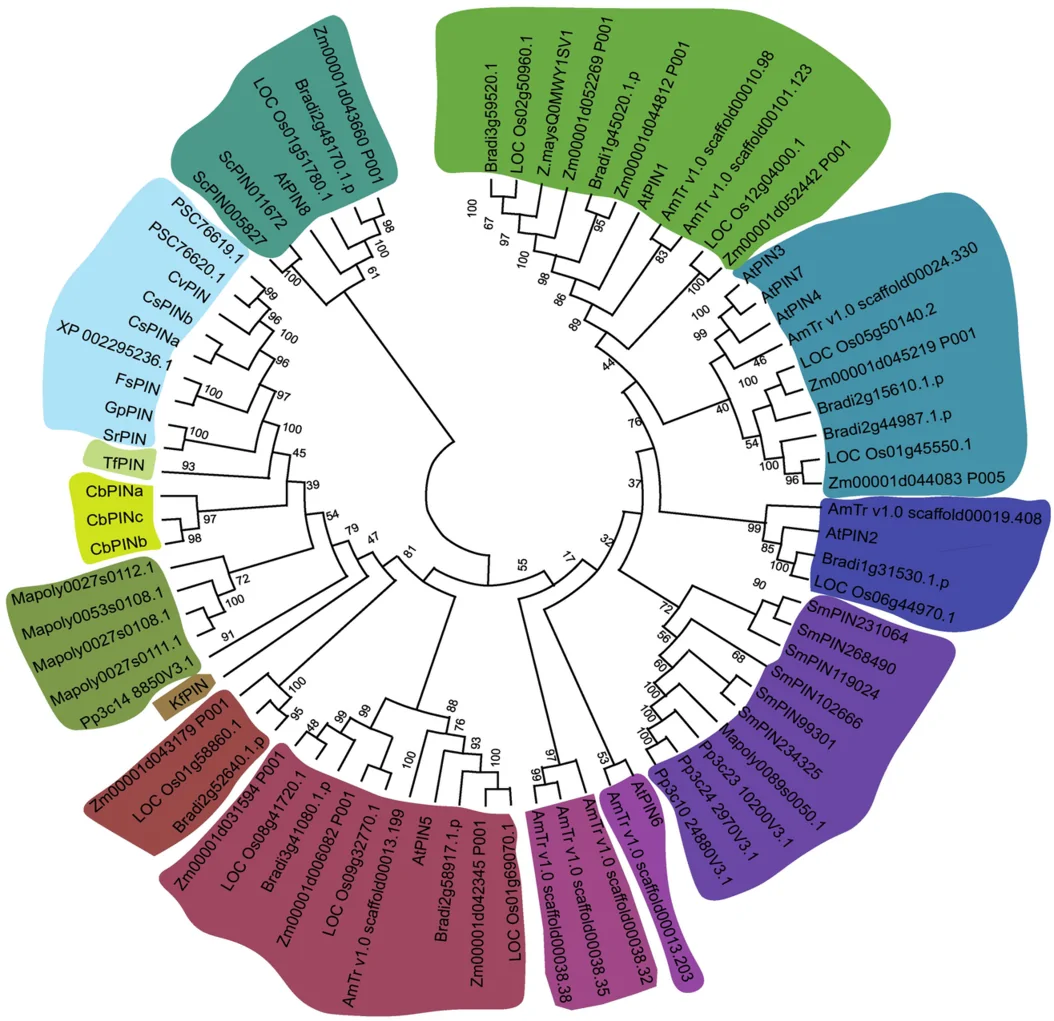
Phylogenetic tree of PIN auxin transporters across plants, algae, fungi, and protists Maximum likelihood phylogenetic tree based on approximately 70 representative PIN protein sequences from 21 species, including both canonical long PINs and short PINs (PIN5‐, PIN6‐, and PIN8‐like). Fourteen distinct clades (Groups 1–14) were recovered with moderate to strong bootstrap support. Short PINs form a distinct clade, consistent with their reduced length and divergent loop architecture. Group 13 includes chlorophyte, diatom, fungal, and protist PIN homologs. Species are color‐coded as follows: black for angiosperms, red for non‐seed plants, and gray for algae and protists. Scale bar indicates substitutions per site.

The resulting phylogeny resolved 14 distinct clades (Groups 1–14), each supported by moderate to strong bootstrap values. Short PINs formed a distinct clade, consistent with their known divergence from canonical long PIN exporters. Chlorophyte PIN homologs clustered within a well‐supported clade (Group 13; bootstrap value 100), comprising sequences from *C. variabilis* and *C. sorokiniana*, together with homologs from the diatom *Fistulifera solaris*, the protist *Trichomonas vaginalis*, and the chytrid fungus *Gonopodya prolifera*. Notably, chlorophyte PINs did not show close phylogenetic affinity to short PINs, supporting the distinction between these lineages.

*Gonopodya prolifera* PIN clustered within this algal‐dominated clade and displayed characteristic features of PIN‐like transporters rather than those of PILS or unrelated transporters. This relationship may reflect an ancient evolutionary connection between aquatic fungi and green algae, potentially involving horizontal gene transfer, although additional analyses would be required to test this possibility.

To determine whether the algal PIN homologs are transcriptionally active, we performed quantitative RT‐PCR on exponentially growing cultures of *C. sorokiniana* and *C. variabilis*, using 18S rRNA as a reference gene (Figure S6A). Both *CsPIN* and *CvPIN* were expressed at low but detectable levels. Melt curve analyses showed single, sharp peaks at approximately 80°C, confirming amplification specificity (Figure S6B, C). These results indicate that the PIN genes in *Chlorella* are transcribed under standard growth conditions.

Despite lineage‐specific exon–intron variation (Figure S7A), all algal PIN‐like proteins retained the characteristic N‐ and C‐terminal transmembrane architecture, consistent with overall structural conservation despite shorter central hydrophilic loops (Figure S7B, C). The shorter hydrophilic loops observed in algal PINs may reflect ancestral forms predating the extended central loops characteristic of land plant PINs, where many polarity and regulatory interaction features are located.

Together, these phylogenetic, structural, and transcriptional data support the presence of PIN‐like proteins across diverse eukaryotic lineages and indicate that chlorophyte PIN homologs are expressed and structurally conserved.

### Docking predicts auxin binding in non‐plant PINs, but they do not mediate transport in oocytes

To explore whether chlorophyte and non‐plant PIN proteins could interact with IAA and transport it, we aligned their sequences against the structurally characterized *A. thaliana* PIN8 (AtPIN8) (Figure S8). Residues known to coordinate auxin binding in AtPIN8 (Ung et al., 2022) were highlighted and compared across PIN proteins from *C. variabilis* (CvPIN), *C. sorokiniana* (CsPIN), *F. solaris* (FsPIN), and *G. prolifera* (GpPIN). Several of these auxin‐binding residues were conserved, particularly in FsPIN and GpPIN, suggesting that these proteins may retain auxin‐binding capacity.

To further test this possibility, we performed molecular docking simulations using IAA as the ligand and AlphaFold2‐predicted structures of each PIN variant as receptors (Figure 4A–D). Docking was guided by conserved amino acids identified in the published AtPIN8 structure (Ung et al., 2022) and revealed favorable predicted binding energies across all tested PINs. FsPIN (−6.7 kcal mol^−1^) and GpPIN (−6.5 kcal mol^−1^) showed the strongest predicted affinities, followed by CvPIN (−5.9 kcal mol^−1^) and CsPIN (−5.4 kcal mol^−1^). Binding energies are summarized in Table S1.

**Figure 4:**
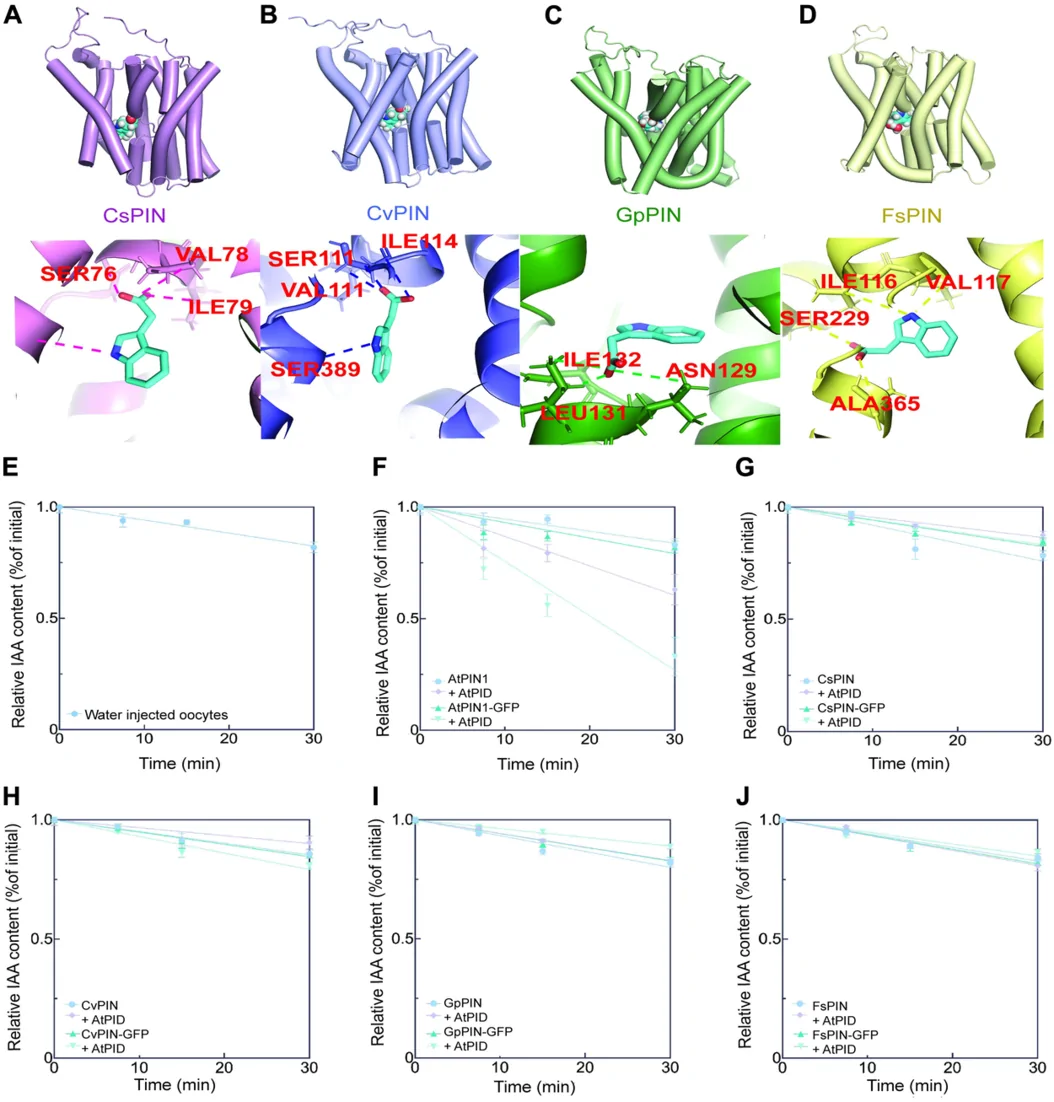
Docking predicts IAA binding in non‐plant PINs, but they do not mediate transport in oocytes **(A**–**D)** Molecular docking of indole‐3‐acetic acid (IAA) to AlphaFold2‐predicted structures of CsPIN (*Chlorella sorokiniana*), CvPIN (*Chlorella variabilis*), FsPIN (*Fistulifera solaris*), and GpPIN (*Gonopodya prolifera*). The bound IAA ligand and predicted hydrogen bonds (dashed lines) are shown, with key residues in the binding pocket highlighted. **(E**–**J)** Time course of [^3^H]‐IAA export in *Xenopus laevis* oocytes expressing PINs either alone or co‐expressed with the *Arabidopsis thaliana* kinase PINOID (PID). **(E)** Water‐injected oocytes lacking recombinant PIN expression served as negative controls. **(F)** Native and GFP‐tagged AtPIN1 show active IAA efflux, enhanced by PID co‐expression. **(G**–**J)** CsPIN, CvPIN, FsPIN, and GpPIN, either alone or co‐expressed with PID, do not mediate detectable IAA export. The initial internal IAA concentration was 1 μM. Data points represent the means ± *SE* (*n* = 10 oocytes per time point).

To further assess structural conservation between chlorophyte PIN homologs and land plant PINs, we performed structural superposition with *A. thaliana* PIN8 using PyMOL (Figure S9). The alignment was carried out using a single PIN8 monomer as the reference and backbone Cα atoms to quantify structural similarity. All homologs retained the overall PIN fold, with Cα RMSD values ranging from 3.15 to 4.57 Å across 154–314 aligned residues, indicating conservation of the core transmembrane architecture despite evolutionary divergence. Detailed alignment statistics and RMSD values are provided in Tables S2 and S3.

To test whether chlorophyte and non‐plant PINs have the capacity to mediate IAA efflux, we expressed them in *Xenopus laevis* oocytes, a standard system for assessing the transport capacity of recombinant proteins (Fastner et al., 2017). Oocytes were injected with capped mRNA encoding AtPIN1, CsPIN, CvPIN, FsPIN, or GpPIN, with or without co‐injection of PID kinase mRNA to promote PIN phosphorylation, plasma membrane targeting, and PIN activation (Figure 4E–J). Injection resulted in robust expression and plasma membrane localization of GFP‐tagged PINs (Figure S11). Water‐injected oocytes served as controls (Figure 4E). Auxin transport was measured by monitoring the uptake and efflux of radiolabeled [^3^H]‐IAA. Oocytes expressing AtPIN1 showed a clear reduction in intracellular [^3^H]‐IAA levels, indicating active efflux, which was further enhanced by PID co‐expression. In contrast, oocytes expressing CsPIN, CvPIN, FsPIN, or GpPIN did not show any significant change in auxin accumulation compared with water‐injected controls, even in the presence of PID.

These findings indicate that, although non‐plant PIN homologs retain conserved residues consistent with auxin binding, they do not mediate detectable auxin transport in *X. laevis* oocytes. This uncoupling of predicted binding capacity from transport activity suggests that PIN homologs in chlorophytes and other non‐streptophyte lineages may have functions distinct from canonical auxin export. Such functions may instead relate to intracellular auxin homeostasis or auxin‐associated processes rather than directional transport.

### Non‐plant PINs show ER localization and limited auxin transport in BY‐2 cells

To assess the subcellular localization and auxin transport properties of algal, protist, and basal fungal PIN homologs, we expressed GFP‐tagged CvPIN, GpPIN, and FsPIN under the estradiol‐inducible XVE system in tobacco BY‐2 cells. This system provides a well‐established heterologous model for studying membrane trafficking, transformation efficiency, and auxin transport (An, 1985; Nagata et al., 1992; Petrášek et al., 2006; Jelínková et al., 2010; Skokan et al., 2019). The resulting transgenic lines were used for confocal imaging and auxin accumulation assays (Petrášek et al., 2006). In the same induced cells, we performed auxin transport assays using radiolabeled 1‐naphthaleneacetic acid ([^3^H]‐NAA), a lipophilic synthetic auxin with high specificity for auxin efflux carriers and a preferred tracer for monitoring carrier‐mediated auxin efflux (Delbarre et al., 1996; Petrášek et al., 2006).

CvPIN‐GFP (in induced XVE::CvPIN‐GFP cells) localized predominantly to the endoplasmic reticulum (ER) in interphase cells and was detected at the cell plate during cytokinesis, without a visible plasma membrane (PM) signal (Figure 5A). GpPIN‐GFP and FsPIN‐GFP showed comparable patterns, localizing to the ER and the cell plate in dividing cells, with weak PM signal in interphase cells (Figure 5B, C). Attempts to generate XVE::CsPIN‐GFP transformants were unsuccessful despite 38 independent transformation attempts (Table S4).

**Figure 5:**
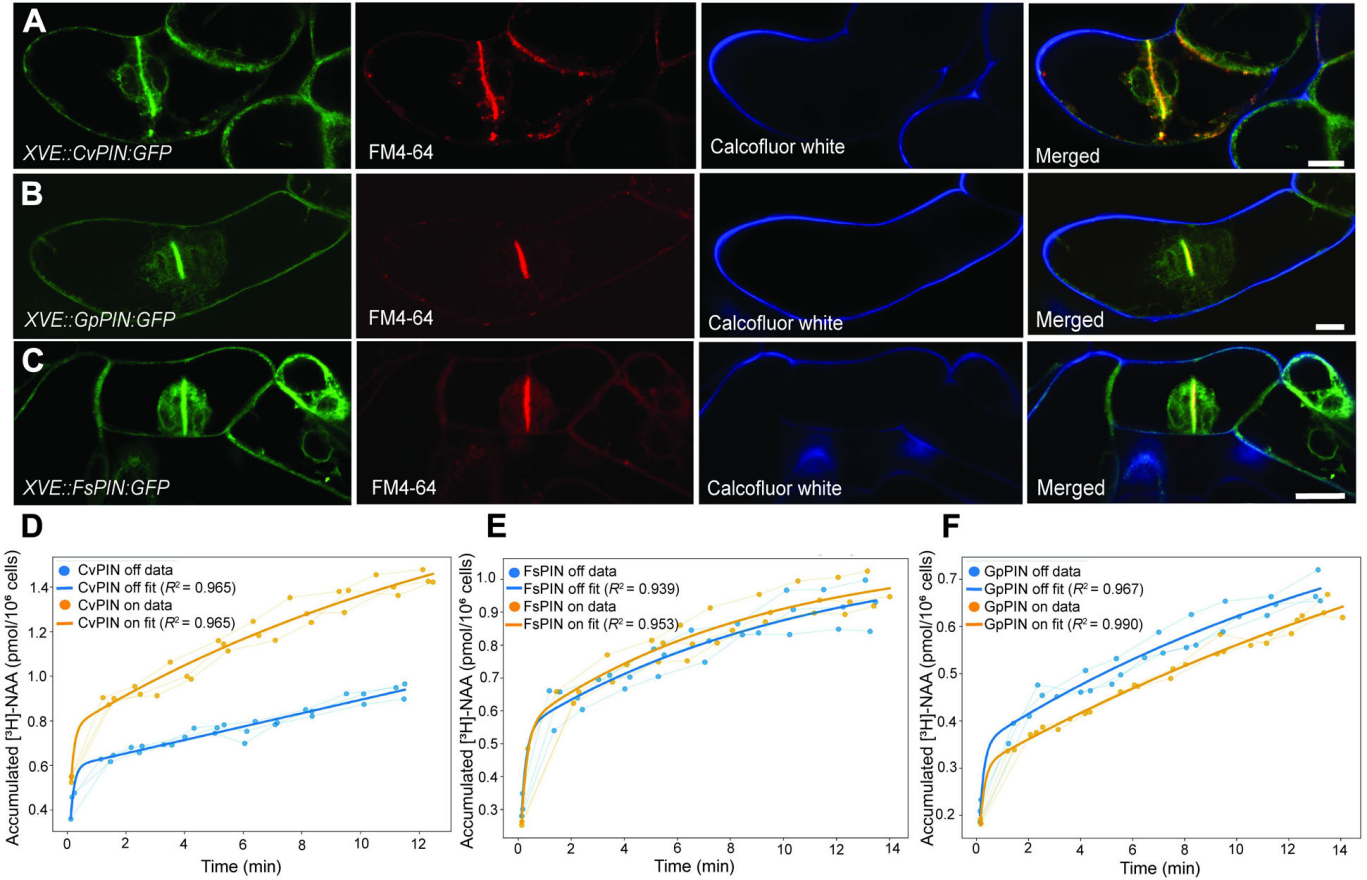
Subcellular localization and auxin transport activity of algal PINs expressed in BY‐2 cells **(A**–**C)** Airyscan confocal images of BY‐2 cells expressing XVE::CvPIN‐GFP **(A)**, XVE::GpPIN‐GFP **(B)**, and XVE::FsPIN‐GFP **(C)**. Shown are GFP fluorescence, FM4‐64 staining of the plasma membrane, Calcofluor White staining of the cell wall, and merged channels. Cells were induced with 1 μM β‐estradiol and imaged 24 h after induction. Scale bars = 10 μm. **(D**–**F)** Auxin accumulation assays in BY‐2 cells expressing the respective PINs. Accumulation of 2 nM [^3^H]‐NAA is shown for non‐induced (PIN off, DMSO‐treated) and induced (PIN on, β‐estradiol‐treated) cells. Individual biological replicates are plotted as circles, with faint lines indicating replicate kinetics. Solid lines represent weighted double‐exponential fits (*Y* = *a*_1_(1 − *e* ^−b1t^) + *a*_2_(1 − *e* ^−b2t^)), with standard deviations (*SD*) used for weighting. Representative fit quality: CvPIN off, *R* ^2^ = 0.966; CvPIN on, *R* ^2^ = 0.974; FsPIN off, *R* ^2^ = 0.939; FsPIN on, *R* ^2^ = 0.951; GpPIN off, *R^2^* = 0.968; GpPIN on, *R* ^2^ = 0.991.

To independently verify ER localization, GFP‐tagged PIN proteins were co‐expressed with the ER marker 35S::HDEL‐mCherry in *Nicotiana benthamiana* epidermal cells (Figure S10). Confocal imaging revealed strong co‐localization of CvPIN, CsPIN, GpPIN, and FsPIN with the ER marker, confirming their predominant localization to the endoplasmic reticulum. Sequence analysis using SignalP‐5.0 did not predict a classical N‐terminal ER signal peptide in any of the chlorophyte PIN homologs, consistent with their multi‐pass transmembrane topology. Importantly, the observed fluorescence displayed a continuous reticulate pattern characteristic of the ER network (Figures 5, S10), rather than punctate aggregates, and was reproducible across independent experiments, making misfolding‐induced ER retention unlikely.

These results support the interpretation that the tested PIN homologs primarily localize to the ER rather than the plasma membrane in this heterologous system. Auxin accumulation assays revealed distinct functional behaviors (Figure 5D–F). CvPIN‐GFP expression resulted in increased accumulation of 1‐NAA (Figure 5D), consistent with enhanced auxin retention, similar to the behavior of CbPINa from the complex charophyte alga *Chara braunii* (Kurtović et al., 2025). FsPIN‐GFP showed no measurable difference between induced and non‐induced cells, indicating no detectable auxin transport activity (Figure 5E). GpPIN‐GFP produced a slight reduction in 1‐NAA accumulation, suggesting very weak auxin efflux activity (Figure 5F).

These results from tobacco cells show that algal, protist, and basal fungal PIN homologs localize predominantly to the endoplasmic reticulum, with limited plasma membrane presence. Expression of CvPIN resulted in increased intracellular auxin accumulation, suggesting enhanced auxin retention and a potential role in intracellular auxin homeostasis. In contrast, the other tested PIN homologs did not show detectable effects on auxin accumulation.

### Non‐plant PINs do not influence auxin‐mediated development in *A. thaliana*

To investigate whether chlorophyte and non‐plant PIN proteins can affect auxin‐regulated development in *A. thaliana*, we analyzed multiple auxin‐dependent processes in transgenic lines individually expressing PINs from green algae (CsPIN, CvPIN), a diatom (FsPIN), and a fungus (GpPIN), driven under the constitutive 35S promoter in a wild‐type (Col‐0) background. We selected transgenic lines confirmed by PCR‐based genotyping and confocal microscopy showing detectable and comparable PIN–GFP expression. Root hair development in these lines was unaffected, with no noticeable differences in root hair length or density compared with wild type (Figure 6A, B). Likewise, vein patterning in cotyledons remained unchanged, and quantification of defective venation patterns revealed no significant deviations from wild‐type architecture (Figure 6C, D). These findings suggest that ectopic expression of chlorophyte and non‐plant PINs does not measurably alter auxin homeostasis, consistent with their lack of detectable IAA transport activity in heterologous systems.

**Figure 6:**
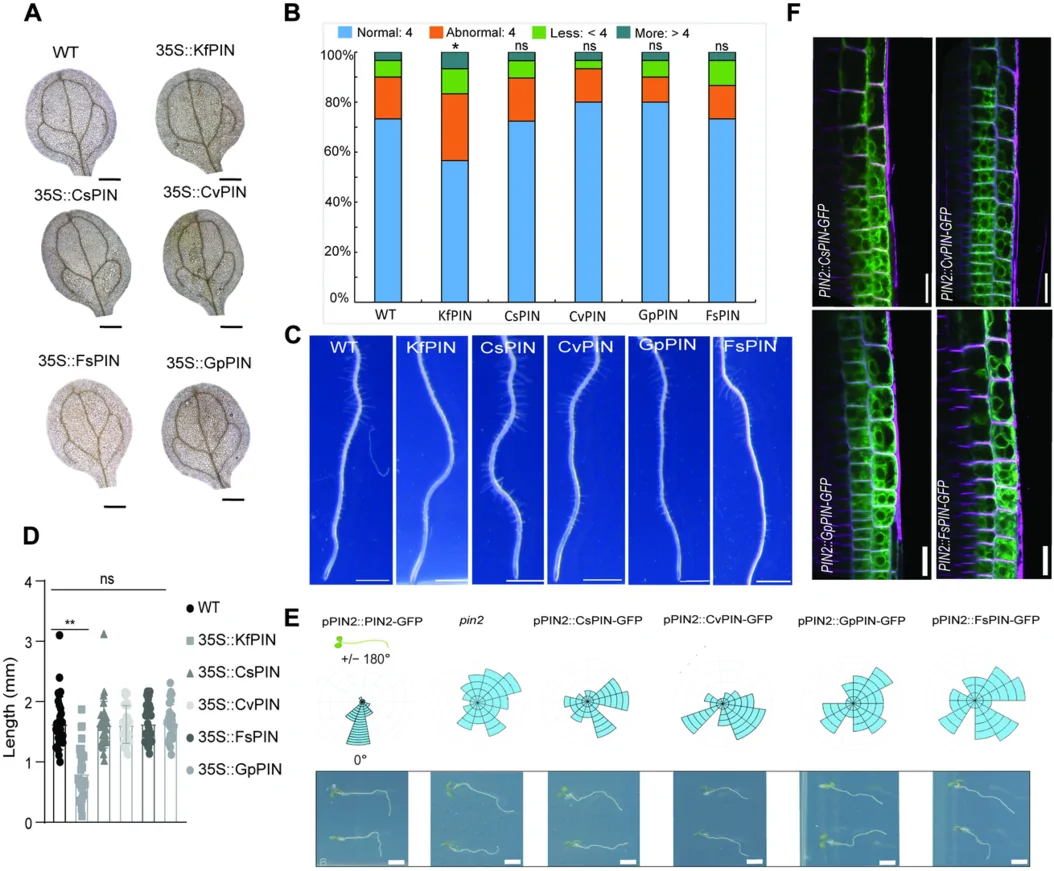
**Functional assessment of non‐plant PINs in** ***Arabidopsis thaliana*** **(A)** Vascular patterning in primary leaves of 9 d‐old *A. thaliana* seedlings showing representative normal and abnormal vein patterns. **(B)** Quantification of vascular pattern phenotypes shown in **(A)**. Venation patterns were categorized as normal or abnormal and compared with wild type using Fisher's exact test. A significant increase in abnormal venation was observed in 35S::KfPIN (*P* < 0.05), whereas other lines showed no significant differences. **(C)** Root hair length in 8 d‐old *A. thaliana* wild type and transgenic lines expressing 35S::KfPIN, 35S::CsPIN, 35S::CvPIN, 35S::GpPIN, and 35S::FsPIN. **(D)** Quantification of root hair lengths shown in **(C)**. Only 35S::KfPIN significantly reduced root hair length compared with wild type (Dunnett's test, *P* < 0.0001). **(E)** Gravitropic root bending in 4 d‐old seedlings of the *pin2* mutant, pPIN2::PIN2 complementation control, and pPIN2::PIN‐expressing lines (CsPIN, CvPIN, GpPIN, and FsPIN) after 90° reorientation. Bending angles were analyzed as absolute values to quantify curvature independently of direction. Transgenic lines showed significantly increased bending compared with wild type (one‐way ANOVA, *P* < 0.0001; Tukey's multiple comparisons test), whereas no significant differences were observed among the transgenic lines. **(F)** Subcellular localization of pPIN2::CsPIN‐GFP, pPIN2::CvPIN‐GFP, pPIN2::FsPIN‐GFP, and pPIN2::GpPIN‐GFP in *Arabidopsis pin2* root epidermal cells, showing mixed localization at the plasma membrane and endoplasmic reticulum. Scale bars = 1 mm **(A)**, 2 mm **(E)**, and 20 μm **(F)**.

To further evaluate whether non‐plant PINs could functionally substitute for endogenous PIN proteins, we individually expressed CsPIN‐GFP, CvPIN‐GFP, FsPIN‐GFP, and GpPIN‐GFP in *A. thaliana*, driven by the Arabidopsis PIN2 promoter (*pPIN2*), and introduced them into the *pin2* mutant background. We then tested their ability to rescue the characteristic *pin2* root gravitropic defect, a hallmark of disrupted PIN‐mediated auxin transport (Luschnig et al., 1998). Following 90° reorientation, seedlings expressing non‐plant PINs showed randomized and inconsistent root bending, similar to the *pin2* mutant, whereas control *pPIN2::AtPIN2‐GFP* complementation lines displayed a strong directional response (Figure 6E).

Confocal microscopy of *pPIN2::CsPIN‐GFP*, *pPIN2::CvPIN‐GFP*, *pPIN2::FsPIN‐GFP*, and *pPIN2::GpPIN‐GFP* lines revealed that all fluorescent fusion proteins localized to both the plasma membrane (PM) and the endoplasmic reticulum (ER) (Figure 6F). This dual localization pattern resembles that observed in transient expression assays (Figure S10) and suggests that subcellular trafficking of chlorophyte and non‐plant PINs differs from that of endogenous PIN2, which localizes predominantly to the PM.

Together, these findings in Arabidopsis confirm dual ER and PM localization of chlorophyte and non‐plant PINs but do not support a role for these proteins in regulating auxin homeostasis or auxin‐mediated development.

## DISCUSSION

Our study shows that chlorophyte algae are auxin‐responsive but do not possess the canonical PIN‐dependent auxin export systems of land plants. Despite lacking this key regulatory framework underlying directional auxin transport in multicellular streptophytes, chlorophytes respond to exogenously applied IAA in a dose‐dependent manner. This indicates that the capacity to sense and respond to auxin predates the emergence of specialized transport and signaling networks that later became central to land plant development.

The biphasic response to auxin, with growth promotion at low concentrations and inhibition at higher concentrations, resembles classical auxin responses in land plants (Gallei et al., 2020). In chlorophytes, however, this effect is primarily mediated through changes in division timing rather than cell expansion, suggesting that auxin regulates proliferation dynamics rather than coordinated tissue growth. The lack of a similar response to tryptophan further distinguishes chlorophytes from streptophyte algae such as *Penium margaritaceum* (Carrillo‐Carrasco et al., 2025), indicating differences in auxin biosynthesis or metabolic integration.

A central implication of our data is that auxin responsiveness can be uncoupled from PIN‐mediated directional export. Chlorophyte cells take up and release IAA through both passive and energy‐dependent processes, yet the PIN homologs tested here do not mediate detectable auxin efflux in *X. laevis* oocytes. This suggests that auxin response mechanisms evolved prior to PIN‐dependent directional transport. Consistent with this, chlorophytes possess auxin synthesis, secretion, and perception pathways despite lacking canonical polar auxin transport systems (Khasin et al., 2018).

Our transport assays further indicate that auxin movement in chlorophytes involves carrier‐mediated components with broader substrate recognition, as both natural IAA and synthetic NAA compete for transport. Similar substrate flexibility for auxin export has been described in land plants (Fukui et al., 2018), and structural studies of AUX1 auxin influx carriers support shared binding mechanisms for diverse auxin analogs (Chen et al., 2025). These findings suggest that auxin transport in chlorophytes relies on less specialized carriers, whose molecular identity remains unknown but most likely differs from canonical PIN and AUX1/LAX transport components of land plants.

The subcellular behavior of non‐plant PIN homologs provides further insight into their functional divergence. Their predominant localization to the endoplasmic reticulum, together with the absence of detectable auxin efflux activity, argues against a role analogous to plasma membrane‐localized PIN exporters of streptophytes. Instead, these proteins may function in intracellular auxin handling, such as compartmentalization or homeostasis. Although structural analyses reveal conservation of the PIN fold and predicted auxin‐binding residues (Ung et al., 2022), this conservation is not coupled to export activity, indicating functional divergence.

Consistent with this, heterologous expression of chlorophyte and non‐plant PINs in *A. thaliana* did not affect auxin‐dependent developmental processes and failed to complement the *pin2* agravitropic root growth phenotype. These results define clear functional limits for non‐streptophyte PIN homologs and indicate that they do not substitute for canonical PIN‐mediated auxin transport in multicellular plant systems.

From an evolutionary perspective, our findings support a stepwise model in which auxin perception and basic transport capacities preceded the emergence of polar auxin transport. PIN‐mediated directional auxin export appears to be a later innovation associated with the streptophyte lineage, where polarized trafficking and tissue‐level coordination evolved (Blázquez et al., 2020; Luschnig and Friml, 2024).

While our data do not support a role for chlorophyte PINs in directional IAA export, these proteins may have broader substrate specificity or transport alternative auxin‐related metabolites. Early‐diverging transporters often exhibit broader substrate specificity, with increased specialization emerging later during evolution through gene duplication and subfunctionalization (Jørgensen et al., 2017). Identifying alternative substrates will require dedicated biochemical or metabolomic approaches. Definitive functional analysis will require genetic approaches, which are currently limited in *Chlorella* species (Kim et al., 2021; Velmurugan and Muthukaliannan, 2022).

## MATERIALS AND METHODS

### Plant materials and growth conditions

*Chlorella sorokiniana* (SAG 211‐8 K), *C. variabilis* (NC64A), and *C. reinhardtii* (CC‐4533) were grown in sterile liquid TAP medium (Gorman and Levine, 1965) under constant light (100 μmol m^−2^ s^−1^) at 25°C with gentle shaking (120 rpm). Cultures were maintained in the exponential phase by subculturing every 3–4 d. For optimal growth, *C. sorokiniana* was also cultivated in BG‐11 medium, whereas *C. variabilis* grew well in Bold's Basal Medium (BBM).

Cells of the tobacco BY‐2 line (*Nicotiana tabacum* L. cv. Bright Yellow 2) (Nagata et al., 1992) were cultured in liquid medium containing 30 g/L sucrose, 4.3 g/L Murashige and Skoog salts (Sigma M5524), 100 mg/L inositol, 1 mg/L thiamine, 0.2 mg/L 2,4‐D, and 200 mg/L KH_2_PO_4_ (pH 5.8). Cultures were maintained in darkness at 27°C on an orbital shaker (Sanyo Gallenkamp, Schoeller Instruments Inc., Prague, Czech Republic; 150 rpm, 32 mm orbit) and subcultured weekly. Stock calli were maintained on the same medium, solidified with 0.6% (w/v) agar, and subcultured monthly.

### Auxin and tryptophan treatments

Indole‐3‐acetic acid (IAA; Sigma‐Aldrich) and L‐tryptophan (Sigma‐Aldrich) were dissolved in ethanol and sterile‐filtered. Algal cultures with an initial cell density of 2 × 10^6^ cells/mL were treated with final concentrations of 0 (mock), 1 μM, 10 μM, and 50 μM IAA, or 10 μM tryptophan. The same volume of ethanol was added to mock controls. Treatments were performed in triplicate, with cell densities normalized across conditions.

### Cell counting and biomass estimation

Cell density and growth were monitored every 24 h from time 0 to Day 4. Cell density was quantified using a Bio‐Rad automated cell counter, and OD_750_ was recorded as a proxy for biomass using a Thermo Scientific plate reader. At least five biological replicates were measured per condition.

### Microfluidic imaging of cell division

The chamber layout was based on previously described microfluidic platforms for *C. reinhardtii*, with minor modifications (Széles et al., 2022). Device fabrication involved two main steps: photolithographic patterning of silicon wafers and casting of polydimethylsiloxane (PDMS) devices. Device designs were created in CorelDRAW and LinkCAD, and chrome photomasks were produced (JD Photodata). Four‐inch silicon wafers were baked at 110°C for 5 min, then spin‐coated with SU8 6005 at 1,500 rpm for 40 s (acceleration 150 rpm/s). After soft‐baking (5 min at 110°C), wafers were exposed to UV light (100 mJ/cm^2^) using an EVG 610 mask aligner, post‐baked for 5 min at 110°C, and developed in SU8 developer. Devices were hard‐baked at 135°C for 5 min. Feature height was approximately 7 μm, as measured by profilometry. Wafers were silanized in vapor‐phase 1H,1H,2H,2H‐perfluorooctyltrichlorosilane (Sigma) for 1 h. PDMS (Sylgard 184; 10:1 ratio) was poured over the molds and cured. Devices were demolded, ports were punched with a sharpened needle, cleaned with adhesive tape, and plasma‐bonded (Harrick Scientific; medium power, 1 min) to isopropanol‐cleaned microscope slides. Bonded devices were baked at 100°C for 1 h. Tubing and needles were inserted for connection to syringe pumps.

### Radiolabeled IAA uptake and auxin export assays

For algal cells, radiolabeled indole‐3‐acetic acid ([^3^H]‐IAA; American Radiolabeled Chemicals) was used at a final concentration of 5 nM, with a specific activity of 20 Ci mmol^−1^. Uptake (import) and efflux (export) assays were performed to assess auxin transport in algal cells at a starting concentration of 2 × 10^6^ cells/mL.

For uptake assays, cells were incubated with [^3^H]‐IAA for 20 min at room temperature. After incubation, cells were collected by filtration and washed five times with cold buffer to minimize surface‐bound [^3^H]‐IAA. Washed cells were then lysed directly in scintillation cocktail, and intracellular radioactivity was quantified using a Hidex liquid scintillation counter.

For efflux assays, cells were first preloaded with [^3^H]‐IAA for 20 min, followed by five washing steps and filtration to remove extracellular radiolabeled IAA. Washed cells were resuspended in fresh medium and incubated for an additional 20 min to allow IAA export. Cells were then separated from the medium by centrifugation, and the resulting supernatants, corresponding to the cell‐free extracellular medium, were collected. Radioactivity in the supernatants was quantified by liquid scintillation counting and used as a measure of exported [^3^H]‐IAA.

Competition assays were performed by pre‐incubating cells with excess unlabeled (“cold”) IAA (500 nM or 10 μM) for 20 min before adding [^3^H]‐IAA. Both uptake and export were then measured as described above to test the specificity of auxin transport. Radioactivity was measured using a Hidex liquid scintillation counter, and data are expressed as percentages relative to untreated control samples. All assays were performed with at least three independent biological replicates.

### Sodium azide treatment and fluorescence microscopy

To inhibit active transport by disrupting cellular energy metabolism, cells were pretreated with 1 mM sodium azide (NaN_3_) for 30 min prior to the addition of [^3^H]‐IAA. Cells were then processed for radiolabeled IAA uptake and export assays as described above.

Cell viability following azide treatment was assessed using fluorescein diacetate (FDA) staining. Cells were loaded into Tulip microfluidic chambers and incubated with FDA at a final concentration of 5 μg/mL for 5–10 min in the dark, followed by immediate imaging. FDA is hydrolyzed by intracellular esterases in metabolically active cells to produce fluorescent fluorescein.

Fluorescence imaging was performed using a Nikon Ti‐E inverted microscope equipped with a 40× objective under identical acquisition settings for all conditions. Cells displaying detectable fluorescence were scored as viable (FDA‐positive), whereas non‐fluorescent cells were considered non‐viable. For each condition, *n* = 60 cells were analyzed. Statistical significance between control and sodium azide‐treated samples was determined using Fisher's exact test. Significance between control and sodium azide‐treated samples was determined using Fisher's exact test.

### RNA extraction and RT‐qPCR analysis

Total RNA was extracted from mid‐log phase algal cultures using the RNeasy Plant Mini Kit (Qiagen), followed by on‐column DNase I treatment (Thermo Fisher Scientific) to remove genomic DNA contamination. cDNA synthesis was carried out using the iScript cDNA Synthesis Kit (Bio‐Rad) according to the manufacturer's instructions. Quantitative real‐time PCR (RT‐qPCR) was performed using SYBR Green Supermix (Bio‐Rad) on a Roche LightCycler 480 Real‐Time PCR System. Reaction setup was automated using a Beckman Coulter Biomek i5 liquid handling robot to ensure consistency and reproducibility. Gene‐specific primers were designed for *CsPIN*, *CvPIN*, and 18S rRNA (reference gene). Relative expression levels were calculated using the ΔΔCt method, and melt curve analysis was performed to verify amplification specificity.

### Phylogenetic analysis of PIN homologs

PIN protein sequences were identified through HMMER3.3 searches against the NCBI non‐redundant (nr) database, using *A. thaliana* PIN proteins as query profiles. To reduce redundancy, sequences were clustered at 45% identity using USEARCH v11. The resulting sequences were aligned in MEGA11 using the ClustalW algorithm. Phylogenetic reconstruction was performed in MEGA11 using three approaches: neighbor‐joining (NJ), maximum parsimony (MP), and maximum likelihood (ML). NJ analysis was conducted using Poisson correction distances with pairwise deletion of gaps. MP analysis was performed with default parameters. The best‐fitting substitution model for ML analysis was determined using jModelTest2. Bootstrap analysis with 1,000 replicates was used to assess the robustness of all phylogenetic trees. All PIN‐like sequences used in the analysis are listed in Table S6.

### Gene structure and protein modeling

Exon–intron structures were retrieved from genome annotations or predicted using Splign. Transmembrane topology was predicted using TMHMM v2.0. Protein structures of *CsPIN*, *CvPIN*, *GpPIN*, and *FsPIN* were predicted using AlphaFold2 (ColabFold implementation). Hydrophilic loop lengths were estimated from sequence alignments. Structural models were visualized using PyMOL.

### Molecular docking and structural superposition

Molecular docking of indole‐3‐acetic acid (IAA) was performed using AutoDock Vina v1.2.0 with PIN protein structures predicted by AlphaFold2. Binding sites were defined based on conserved residues aligned to *A. thaliana* PIN8, as previously described (Ung et al., 2022). Docking grids were centered on these conserved regions, with affinity grids automatically generated using 4.0 Å padding and a grid spacing of 0.375 Å. Each docking simulation was run with 50 independent runs and 25 million energy evaluations to ensure thorough conformational sampling. Binding affinities were extracted from the top‐ranked binding poses, and protein–ligand complexes were visualized using PyMOL (Schrödinger, LLC). Docking results were further analyzed and inspected using PMV v1.5.7.

Structural superposition of PIN homologs was performed using PyMOL. Predicted structures of CsPIN, CvPIN, FsPIN, and GpPIN were aligned to a single monomer of *A. thaliana* PIN8 using the PyMOL “super” command. Alignments were performed using backbone Cα atoms. Root‐mean‐square deviation (RMSD) values and the number of aligned atoms were calculated for each comparison.

### Vector construction and gene transformation

To generate constructs for genetic complementation in *A. thaliana*, coding sequences of CsPIN, CvPIN, FsPIN, and GpPIN were fused with GFP within the hydrophilic loop. Each CDS–GFP fusion was cloned into the Gateway entry vector pDONR221 or pDONR P4‐P1r, while a 1.4 kb PIN2 promoter fragment was cloned separately into pDONR vectors. For constitutive expression, the CaMV 35S promoter was cloned into pDONR P4‐P1r. Final constructs were assembled using Multisite Gateway LR recombination into the destination vector pB7m24GW.3. Additionally, CDS–GFP fusions of CvPIN, CsPIN, FsPIN, and GpPIN were cloned into the Gateway‐compatible pMDC7 vector to generate XVE‐inducible expression constructs. Arabidopsis transformation was performed using the floral dip method with *Agrobacterium tumefaciens* GV3101. Transgenic seedlings were selected on Arabidopsis medium (AM) supplemented with Basta (glufosinate ammonium) at 50 mg/L.

GFP‐tagged PIN constructs (CvPIN‐GFP, CsPIN‐GFP, FsPIN‐GFP, and GpPIN‐GFP) were cloned into the XVE‐inducible vector pMDC7 via Gateway cloning. Constructs were introduced into *Nicotiana tabacum* BY‐2 cells by *Agrobacterium*‐mediated transformation according to An (1985), as modified by Petrášek et al. (2006). Transformed calli were selected on hygromycin (50 mg/L), and inducible expression was triggered by the addition of 10 μM β‐estradiol. Confocal imaging was performed 24 h post‐induction using a Leica SP8 microscope.

### Gravitropic bending assay

Arabidopsis seedlings were grown vertically on square agar plates under long‐day conditions (16 h light/8 h dark) for 4 d. To induce a gravitropic response, the plates were rotated 90° and incubated vertically for an additional 24 h. After gravistimulation, images of the seedlings were taken, and the bending angles of root tips were measured using ImageJ software.

### Confocal imaging

Confocal microscopy of Arabidopsis was performed on a Zeiss LSM 800 inverted microscope. GFP was excited at 488 nm and emission collected at 500–550 nm; FM4‐64 was excited at 561 nm and emission collected at 670–740 nm.

Confocal imaging was performed using Zeiss LSM 880 or LSM 900 microscopes equipped with Airyscan detectors and a 40× C‐Apochromat 1.2 NA water‐immersion objective. PMs were stained with FM4‐64 (2 μM; Thermo Fisher Scientific) and cell walls with Calcofluor White (1 μg/mL; Merck) for 10 min. Excitation/emission windows were: GFP (488 nm/493–563 nm), FM4‐64 (561 nm/588–691 nm), and Calcofluor White (405 nm/450–473 nm). Airyscan processing was carried out using ZEN Black (LSM 880) or ZEN Blue (LSM 900).

### Oocyte auxin transport assay

*Xenopus laevis* oocyte efflux experiments were performed as previously described (Fastner et al., 2017). For cRNA synthesis, the mMESSAGE mMACHINE™ SP6 Transcription Kit and the MEGAclear Purification of Transcription Reactions Kit (both Thermo Fisher Scientific) were used according to the manufacturer's instructions. One day after surgery, oocytes were injected with 150 ng/μL PIN cRNA, with or without 75 ng/μL kinase cRNA. After 4 d, oocytes were injected with [^3^H]‐IAA (25 Ci mmol^−1^; RC Tritec) to achieve an internal IAA concentration of 1 μM, defined as 100%. After the indicated time points, residual radioactivity in individual oocytes was measured by liquid scintillation counting. Values are expressed relative to the initial 100%. Each time point represents the means ± *SEM* of 10 oocytes.

All *X. laevis* procedures complied with institutional and national guidelines and were approved by the relevant ethics committee.

### Auxin transport assays in BY‐2 cells

Expression of XVE::CvPIN‐GFP, XVE::FsPIN‐GFP, and XVE::GpPIN‐GFP was induced by the addition of 1 μM β‐estradiol at the beginning of the subculture interval. Auxin accumulation assays were performed according to Petrášek et al. (2006). After 48 h of induction, cells were equilibrated in uptake buffer, and cell densities were determined using a Fuchs–Rosenthal hemocytometer. Radiolabeled auxin ([^3^H]‐NAA; 20 Ci mmol^−1^; American Radiolabeled Chemicals) was added, and 0.5 mL aliquots of cell suspension were collected by rapid filtration onto cellulose filters (22 mm diameter). Filters containing cell material were transferred to scintillation vials, extracted with UV‐grade ethanol for 30 min, and radioactivity was quantified using a Packard Tri‐Carb 2900TR scintillation counter.

### Imaging of *N. benthamina* leaves

Transiently transformed *N. benthamiana* leaves, infiltrated with *Agrobacterium tumefaciens* GV3101 (Clough and Bent, 1998), were imaged 2 d post‐transformation using a Zeiss LSM 910 laser scanning confocal microscope. Samples were mounted on glass slides prior to imaging. Laser wavelengths of 488 nm and 561 nm were used to excite GFP and mCherry fluorophores, respectively, and signals were detected using a MA‐PMT detector.

### Data processing and kinetics analysis

Accumulation values were converted from disintegrations per minute (DPM) to pmol per 10^6^ cells after correction for tritium decay and suspension density. Data points yielding undefined or non‐numeric values, for example, due to division by zero during normalization, were excluded from further analysis. Kinetic modeling was performed using a double‐exponential saturation function, *Y*(*t*) = *a*_1_(1 − exp(−*b*_1_ *t*)) + *a*_2_(1 − exp(−*b*_2_ *t*), implemented in Python. Weighted nonlinear least‐squares fitting was performed using standard deviations as weights. Missing or zero standard deviations were replaced using a hierarchical fallback strategy consisting of the median standard deviation within the corresponding group, the global standard deviation, or a minimum value of 0.01. Initial parameter estimates were derived from observed maxima, with bounds set to ([0, 0, 0, 0], [∞, 10.0, ∞, 1.0]). Fit quality was assessed using *R* ^2^. Plots were generated using Matplotlib.

### Generative AI usage statement

Generative AI tools were used exclusively for language editing and improvement of readability. All scientific content, interpretation, and conclusions were generated and verified by the authors.

## CONFLICTS OF INTEREST

The authors declare no conflicts of interest.

## AUTHOR CONTRIBUTIONS

Conceptualization, A.S. and J.F.; Methodology, A.S., D.M., S.K.‐A., Y.Z., J.M., K.K., J.P., P.K., and A.B.; Investigation, A.S., S.K.‐A., Y.Z., J.M., K.K., P.K., and A.B.; Writing original draft, A.S. and J.F.; Writing review and editing, A.S., S.K.‐A., U.Z.H., J.P., and J.F.; Funding acquisition and resources, A.S., U.Z.H., J.P., and J.F.; Supervision, J.F. All authors have read and approved the contents of this paper.

## Supporting information

Additional Supporting Information may be found online in the supporting information tab for this article: http://onlinelibrary.wiley.com/doi/10.1111/jipb.70309/suppinfo

**Figure S1.** Growth of green algae under auxin and tryptophan treatments
**Figure S2.** High auxin concentration inhibits cell division in Tulip chamber imaging
**Figure S3.** Time course of IAA uptake in *Chlamydomonas reinhardtii*
**Figure S4.** Competitive inhibition of auxin uptake and export by excess unlabeled NAA
**Figure S5.** FDA staining of control and sodium azide‐treated cells
**Figure S6.** RT‐qPCR validation of *CsPIN* and *CvPIN* expression
**Figure S7.** Gene structure, secondary structure, and 3D models of PIN proteins
**Figure S8.** Conservation and functional residue annotation in PIN protein alignment
**Figure S9.** Structural superposition of PIN homologs with *Arabidopsis thaliana* PIN8
**Figure S10.** Co‐localization of chlorophyte and non‐plant PIN homologs with an ER marker
**Figure S11.** Representative confocal images of oocytes expressing GFP‐tagged PIN homologs
**Table S1.** Predicted binding energies of auxin (IAA) to PIN proteins from *Chlorella*, *Gonopodya prolifera*, and *Fistulifera solaris*
**Table S2.** Structural superposition using all atoms (PyMOL super)
**Table S3.** Backbone structural comparison using Cα atoms
**Table S4.** Summary of transformation outcomes for non‐plant PIN constructs in tobacco BY‐2 cells
**Table S5.** Primer sequences used for qPCR, cloning, and construct generation

**Video S1.** Time‐lapse imaging of *Chlamydomonas reinhardtii* cell division in Tulip microfluidic chambers under mock conditions

**Video S2.** Time‐lapse imaging of *Chlamydomonas reinhardtii* cells under 50 μM IAA treatment
