# Supplementary material for: Auxin response and PIN‐mediated transport in chlorophyte algae
Source: J Integr Plant Biol. 2026 Jun 10;68(11):3947–61. doi: 10.1111/jipb.70309 (PMC13725606; doi:10.1111/jipb.70309)
Supplement: Supplementary file 1 [file JIPB-68-3947-s003.docx]

**SUPPLEMENTAL DATA

SUPPLEMENTARY FIGURES**

**Figure S1. Growth of green algae under auxin and tryptophan treatments.**

(**A–C**) Growth curves of *Chlorella sorokiniana* (**A**), *Chlorella variabilis* (**B**), and *Chlamydomonas reinhardtii* (**C**) from day 0 to day 4. Cultures were treated with mock, 1 µM, 10 µM, or 50 µM IAA, or 10 µM tryptophan. Initial cell density was 2 × 10⁶ cells/mL. (**D–F**) Optical density (OD₇₅₀) measurements over 4 days for the same species and treatments as in (**A–C**), with a starting OD₇₅₀ of 0.2 on day 0.

**Figure S2. High auxin concentration inhibits cell division in Tulip chamber imaging.**

(**A**) Representative images of cells in Tulip microfluidic chambers at time 0 and after 9 h of imaging under mock and 50 µM IAA treatment. Yellow star indicates a dividing cell in the mock condition. No cell divisions were observed in the presence of 50 µM IAA. Scale bar = 10 µm. (**B**) Quantification of division timing in individual cells. Each dot represents one cell. Mock-treated cells divided between 510–570 min, whereas no divisions were observed in 50 µM IAA-treated cells during the imaging period (630 min). Imaging was terminated at 630 min; cells without observed division were therefore recorded at this time point. Statistical comparison was performed using a Mann–Whitney U test (*P* < 0.0001).

**Figure S3.** **Time course of IAA uptake in *Chlamydomonas reinhardtii*.**

IAA uptake was measured as radioactivity (dpm) at the indicated time points (0–20 min). Gray lines represent individual biological replicates, whereas the black line with error bars shows the mean ± *SD*. Uptake increased rapidly during the first 10 min and then approached a plateau. The dashed vertical line at 10 min marks the approximate onset of saturation, and the shaded region from 10–20 min indicates the plateau phase during which uptake levels remained relatively stable. Mean uptake values increased from 31.79 ± 29.17 dpm at 0 min to 363.61 ± 128.63 dpm at 2 min, 554.02 ± 62.14 dpm at 5 min, and 953.50 ± 161.82 dpm at 10 min, after which uptake remained relatively stable (895.35 ± 107.97 dpm at 15 min and 1,000.67 ± 181.50 dpm at 20 min). Statistical differences between time points were evaluated using one-way ANOVA (*F* = 116.87, *P* = 2.1 × 10⁻³¹).

**Figure S4**. **Competitive inhibition of auxin uptake and export by excess unlabeled NAA**.

**(A)** Net [³H]-IAA accumulation (% of control) after NAA treatment in the presence of 500 nM or 10 µM unlabeled NAA. Excess unlabeled NAA significantly reduced auxin uptake at 10 µM compared with 500 nM (Welch’s *t*-test, two-tailed, *t* = 2.918, *df* = 3.640, *P* = 0.0486; *n* = 3). **(B)** Net [³H]-IAA accumulation (% of control) after NAA treatment, measured as export into the medium under the same conditions. Auxin export was significantly reduced at 10 µM compared with 500 nM unlabeled NAA (Welch’s *t*-test, two-tailed, *t* = 4.879, *df* = 3.017, *P* = 0.0162; *n* = 3). Data are presented as mean ± *SEM*.

**Figure S5. FDA staining of control and sodium azide-treated cells.**

(**A**) Representative fluorescence images of control and sodium azide-treated cells trapped in a Tulip microfluidic chamber. FDA fluorescence indicates metabolically active cells. (**B**) Quantification of FDA-positive cells. Bars represent the percentage of fluorescent cells (control: 52/60; sodium azide: 22/60; n = 60 cells per condition). Sodium azide treatment significantly reduced the proportion of FDA-positive cells (Fisher’s exact test, **** *P* < 0.0001).

**Figure S6. RT-qPCR validation of CsPIN and CvPIN expression.**

**(A)** ΔCt analysis normalized to 18S rRNA revealed significantly higher PIN expression in *Chlorella sorokiniana* compared with *Chlorella variabilis* (unpaired *t*-test, *P* = 0.03). **(B, C)** Melt curves from RT-qPCR of CsPIN and CvPIN showing single dissociation peaks between 60°C and 95°C, confirming specific amplification.

**Figure S7.** **Gene structure, secondary structure, and 3D models of PIN proteins.**

**(A)** Exon-intron organization of *Arabidopsis thaliana* PIN1 and PIN8 compared with non-plant PINs: CvPIN, CsPIN, GpPIN, and FsPIN. **(B)** Predicted secondary structures of CsPIN, CvPIN, GpPIN, and FsPIN compared with AtPIN1 and AtPIN8, generated using DeepTMHMM. **(C)** Predicted 3D protein structures of PINs modeled by AlphaFold. Colors indicate protein regions: green, N-terminus; blue, C-terminus; and pink, hydrophilic loop.

**Figure** S**8. Conservation and functional residue annotation in PIN protein alignment.**

Conserved residues are shown in a color-scale gradient, with dark purple indicating perfect conservation. Functionally important residues are highlighted: red for IAA hydrophobic recognition, green for support site residues, and orange for central prolines in the crossover motif.

**Figure S9. Structural superposition of PIN homologs with *Arabidopsis* PIN8.**

The predicted structures of PIN homologs were superimposed onto a single monomer of *Arabidopsis* PIN8 using PyMOL and displayed in cartoon representation. PIN8 is shown in pastel grey, CvPIN in pastel pink, CsPIN in pastel violet, GpPIN in pastel green, and FsPIN in pastel yellow. Proteins are shown in the same orientation after structural alignment.

**Figure S10. Co-localization of chlorophyte and non-plant PIN homologs with an ER marker.**

GFP-tagged CvPIN, CsPIN, GpPIN, and FsPIN were transiently expressed in *Nicotiana benthamiana* epidermal cells together with the ER marker 35S::HDEL-mCherry. Each row shows GFP signal (PIN-GFP), ER marker (HDEL-mCherry), and merged images. Scale bars = 5 µm.

**Figure S11.** Representative confocal images of *Xenopus laevis* oocytes expressing GFP-tagged CsPIN **(A)**, CvPIN **(B)**, GpPIN **(C)**, and FsPIN **(D)**, showing localization at the plasma membrane. Scale bar = 100 µm

**SUPPLEMENTARY TABLES**

**Table S1.** Predicted binding energies of auxin (IAA) to PIN proteins from *Chlorella*, *Gonopodya prolifera*, and *Fistulifera solaris*.

**Table S2.** Structural superposition using all atoms (PyMOL super)

| **Protein** | **RMSD vs PIN8 monomer (Å)** | **Aligned atoms** |
| --- | --- | --- |
| CsPIN | 3.236 | 927 |
| CvPIN | 3.988 | 1762 |
| FsPIN | 3.819 | 1747 |
| GpPIN | 4.755 | 1871 |

**Table S3.** Backbone structural comparison using Cα atoms.

| **Protein** | **Cα RMSD vs PIN8 monomer (Å)** | **Aligned Cα atoms** |
| --- | --- | --- |
| CsPIN | 3.146 | 154 |
| CvPIN | 3.926 | 300 |
| FsPIN | 3.716 | 299 |
| GpPIN | 4.569 | 314 |

**Table S4.** Summary of transformation outcomes for non-plant PIN constructs in tobacco BY-2 cells.

**Table S5.** Primer sequences used for qPCR, cloning, and construct generation.

| **Primer Name** | **Sequence (5′ → 3′)** | **Purpose** |
| --- | --- | --- |
| **qPCR primers** |  |  |
| *Chlorella sorokiniana*18SF | CATCTAAGGGCATCACAGAC | Reference gene (Cs) |
| *Chlorella sorokiniana*18SR | TGATTCCGGTAACGAACGAG | Reference gene (Cs) |
| *Chlorella variabilis*18SF | CCTGCGGCTTAATTTGACTC | Reference gene (Cv) |
| *Chlorella variabilis*18SR | ATCAACCTGACAAGGCAACC | Reference gene (Cv) |
| CsPIN_qPCR_F | GCGCTGAGCAAGACCTATAA | CsPIN expression |
| CsPIN_qPCR_R | ATGAACTCCAGCCAGATGATG | CsPIN expression |
| CvPIN_qPCR_F | GCTGCGCCTAGTGATGAA | CvPIN expression |
| CvPIN_qPCR_R | GTGCCAGGGTTGAGGTATC | CvPIN expression |
| **XVE cloning primers** |  | For Gateway-compatible XVE constructs |
| CsPIN_XVE_FOR | CTCACAACAAGCCCAATGCGCGTCTTCAACATTTTCC | CsPIN Gateway XVE cloning |
| CsPIN_XVE_REV | ACGGAGCGGGGATCCTCAGCACGTGCTGGGC | CsPIN Gateway XVE cloning |
| CvPIN_XVE_FOR | CTCACAACAAGCCCAATGTGGGAGTTCCAGGACGT | CvPIN Gateway XVE cloning |
| CvPIN_XVE_REV | ACGGAGCGGGGATCCTCAAGCGTCCCAGGC | CvPIN Gateway XVE cloning |
| FsPIN_XVE_FOR | CTCACAACAAGCCCAATGGTGTACAAGTACATCAGCG | FsPIN Gateway XVE cloning |
| FsPIN_XVE_REV | ACGGAGCGGGGATCCTCAGATGAGGTAGAGC | FsPIN Gateway XVE cloning |
| GpPIN_XVE_FOR | CTCACAACAAGCCCAATGTCGGACCCCCAGG | GpPIN Gateway XVE cloning |
| GpPIN_XVE_REV | ACGGAGCGGGGATCCTCACTTCAGCCGGAG | GpPIN Gateway XVE cloning |
| **Gateway cloning primers (pENTR221)** |  |  |
| CvPIN_221_fw | ggggacaagtttgtacaaaaaagcaggcttcATGTGGGAGTTCCAGGATGTGC | Entry clone (CvPIN) |
| CvPIN_221_rev | ggggaccactttgtacaagaaagctgggttTCAAGCATCCCAAGCAACACAA | Entry clone (CvPIN) |
| CsPIN_221_fw | ggggacaagtttgtacaaaaaagcaggcttcATGAGGGTGTTCAACATCTTCC | Entry clone (CsPIN) |
| CsPIN_221_rev | ggggaccactttgtacaagaaagctgggttTCAACAAGTAGACGGCACCTTA | Entry clone (CsPIN) |
| FsPIN_221_fw | ggggacaagtttgtacaaaaaagcaggcttcATGGTGTACAAGTACATCAGCG | Entry clone (FsPIN) |
| FsPIN_221_rev | ggggaccactttgtacaagaaagctgggttTCAGATGAGGTAAAGCTCGAAG | Entry clone (FsPIN) |
| GpPIN_221_fw | ggggacaagtttgtacaaaaaagcaggcttcATGTCTGATCCTCAAGCTGCTG | Entry clone (GpPIN) |
| GpPIN_221_rev | ggggaccactttgtacaagaaagctgggttTCACTTGAGCCTGAGGGTATCG | Entry clone (GpPIN) |
| **GFP fusion primers** |  | For C-terminal GFP fusions |
| CvPIN_GFP_fw | CATGGACGAGCTGTACAAGGCTCCTCCACAACCTCAAGGA | CvPIN-GFP fusion |
| CvPIN_GFP_rev | CCTCGCCCTTGCTCACCATTTGTTGTTGCTCAGGCAAAGC | CvPIN-GFP fusion |
| CsPIN_GFP_fw | CATGGACGAGCTGTACAAGACCGATGGATTTGGAGAGCAC | CsPIN-GFP fusion |
| CsPIN_GFP_rev | CCTCGCCCTTGCTCACCATAAGAAGAGGAGCAGATCCACC | CsPIN-GFP fusion |
| FsPIN_GFP_fw | CATGGACGAGCTGTACAAGCCTCACGAGATCAACAAGGTG | FsPIN-GFP fusion |
| FsPIN_GFP_rev | CCTCGCCCTTGCTCACCATCTCAGTAGATTCTTTCTCAGC | FsPIN-GFP fusion |
| GpPIN_GFP_fw | CATGGACGAGCTGTACAAGCCTGCTGATATTACCGCTATT | GpPIN-GFP fusion |
| GpPIN_GFP_rev | CCTCGCCCTTGCTCACCATAGCAGGATCAGCAGAAGATCC | GpPIN-GFP fusion |

**Table S6. Amino acid sequences of PIN proteins used in this study.**

Amino acid sequences of PIN proteins from representative species across the green lineage and related taxa used for sequence comparison and phylogenetic analyses in this study. The dataset includes PIN proteins from Arabidopsis thaliana (AtPIN1–AtPIN8; angiosperm, eudicot), Amborella trichopoda (AmTr_v1.0_scaffold00010.98, AmTr_v1.0_scaffold00013.199, etc.; basal angiosperm), Thalictrum flavum (TfPIN; angiosperm, eudicot), Brachypodium distachyon (Bradi1g31530.1.p, Bradi1g45020.1.p, etc.; monocot grass), Oryza sativa (LOC_Os02g50960.1, LOC_Os12g04000.1, etc.; monocot grass), Zea mays (Zm00001d045219_P001; monocot grass), Selaginella moellendorffii (SmPIN99301; lycophyte), Selaginella rupestris (SrPIN; lycophyte), Physcomitrium patens (Pp3c23_10200V3.1; moss), Marchantia polymorpha (Mapoly0027s0111.1; liverwort), Klebsormidium flaccidum (KfPIN; charophyte green alga), Chlorella variabilis (CvPIN; chlorophyte green alga), Chlorella sorokiniana (CsPINa, CsPINb; chlorophyte green alga), Fistulifera solaris (FsPIN; diatom), and Gonopodya sp. (GpPIN; fungus). Sequence identifiers correspond to the original database annotations.

**>AtPIN1**

MITAADFYHVMTAMVPLYVAMILAYGSVKWWKIFTPDQCSGINRFVALFAVPLLSFHFIAANNPYAMNLRFLAADSLQKVIVLSLLFLWCKLSRNGSLDWTITLFSLSTLPNTLVMGIPLLKGMYGNFSGDLMVQIVVLQCIIWYTLMLFLFEYRGAKLLISEQFPDTAGSIVSIHVDSDIMSLDGRQPLETEAEIKEDGKLHVTVRRSNASRSDIYSRRSQGLSATPRPSNLTNAEIYSLQSSRNPTPRGSSFNHTDFYSMMASGGGRNSNFGPGEAVFGSKGPTPRPSNYEEDGGPAKPTAAGTAAGAGRFHYQSGGSGGGGGAHYPAPNPGMFSPNTGGGGGTAAKGNAPVVGGKRQDGNGRDLHMFVWSSSASPVSDVFGGGGGNHHADYSTATNDHQKDVKISVPQGNSNDNQYVEREEFSFGNKDDDSKVLATDGGNNISNKTTQAKVMPPTSVMTRLILIMVWRKLIRNPNSYSSLFGITWSLISFKWNIEMPALIAKSISILSDAGLGMAMFSLGLFMALNPRIIACGNRRAAFAAAMRFVVGPAVMLVASYAVGLRGVLLHVAIIQAALPQGIVPFVFAKEYNVHPDILSTAVIFGMLIALPITLLYYILLGL

>AtPIN2

MITGKDMYDVLAAMVPLYVAMILAYGSVRWWGIFTPDQCSGINRFVAVFAVPLLSFHFISSNDPYAMNYHFLAADSLQKVVILAALFLWQAFSRRGSLEWMITLFSLSTLPNTLVMGIPLLRAMYGDFSGNLMVQIVVLQSIIWYTLMLFLFEFRGAKLLISEQFPETAGSITSFRVDSDVISLNGREPLQTDAEIGDDGKLHVVVRRSSAASSMISSFNKSHGGGLNSSMITPRASNLTGVEIYSVQSSREPTPRASSFNQTDFYAMFNASKAPSPRHGYTNSYGGAGAGPGGDVYSLQSSKGVTPRTSNFDEEVMKTAKKAGRGGRSMSGELYNNNSVPSYPPPNPMFTGSTSGASGVKKKESGGGGSGGGVGVGGQNKEMNMFVWSSSASPVSEANAKNAMTRGSSTDVSTDPKVSIPPHDNLATKAMQNLIENMSPGRKGHVEMDQDGNNGGKSPYMGKKGSDVEDGGPGPRKQQMPPASVMTRLILIMVWRKLIRNPNTYSSLFGLAWSLVSFKWNIKMPTIMSGSISILSDAGLGMAMFSLGLFMALQPKIIACGKSVAGFAMAVRFLTGPAVIAATSIAIGIRGDLLHIAIVQAALPQGIVPFVFAKEYNVHPDILSTAVIFGMLVALPVTVLYYVLLGL

>AtPIN3

MISWHDLYTVLTAVIPLYVAMILAYGSVRWWKIFSPDQCSGINRFVAIFAVPLLSFHFISTNNPYAMNLRFIAADTLQKIIMLSLLVLWANFTRSGSLEWSITIFSLSTLPNTLVMGIPLLIAMYGEYSGSLMVQIVVLQCIIWYTLLLFLFEFRGAKMLIMEQFPETAASIVSFKVESDVVSLDGHDFLETDAEIGDDGKLHVTVRKSNASRRSFCGPNMTPRPSNLTGAEIYSLSTTPRGSNFNHSDFYNMMGFPGGRLSNFGPADMYSVQSSRGPTPRPSNFEENCAMASSPRFGYYPGGGAGSYPAPNPEFSSTTTSTANKSVNKNPKDVNTNQQTTLPTGGKSNSHDAKELHMFVWSSNGSPVSDRAGLNVFGGAPDNDQGGRSDQGAKEIRMLVPDQSHNGETKAVAHPASGDFGGEQQFSFAGKEEEAERPKDAENGLNKLAPNSTAALQSKTGLGGAEASQRKNMPPASVMTRLILIMVWRKLIRNPNTYSSLIGLIWALVAFRWHVAMPKIIQQSISILSDAGLGMAMFSLGLFMALQPKLIACGNSVATFAMAVRFLTGPAVMAVAAIAIGLRGDLLRVAIVQAALPQGIVPFVFAKEYNVHPAILSTGVIFGMLIALPITLVYYILLGL

>AtPIN4

MITWHDLYTVLTAVVPLYVAMILAYGSVQWWKIFSPDQCSGINRFVAIFAVPLLSFHFISTNDPYAMNFRFVAADTLQKIIMLVLLALWANLTKNGSLEWMITIFSLSTLPNTLVMGIPLLIAMYGTYAGSLMVQVVVLQCIIWYTLLLFLFEYRGAKLLIMEQFPETGASIVSFKVESDVVSLDGHDFLETDAEIGNDGKLHVTVRKSNASRRSLMMTPRPSNLTGAEIYSLSSTPRGSNFNHSDFYSVMGFPGGRLSNFGPADLYSVQSSRGPTPRPSNFEENNAVKYGFYNNTNSSVPAAGSYPAPNPEFSTGTGVSTKPNKIPKENQQQLQEKDSKASHDAKELHMFVWSSSASPVSDVFGGGAGDNVATEQSEQGAKEIRMVVSDQPRKSNARGGGDDIGGLDSGEGEREIEKATAGLNKMGSNSTAELEAAGGDGGGNNGTHMPPTSVMTRLILIMVWRKLIRNPNTYSSLIGLIWALVAYRWHVAMPKILQQSISILSDAGLGMAMFSLGLFMALQPKIIACGNSVATFAMAVRFITGPAIMAVAGIAIGLHGDLLRIAIVQAALPQGIVPFVFAKEYNVHPTILSTGVIFGMLIALPITLVYYILLGL

>AtPIN5

MINCGDVYKVIEAMVPLYVALILGYGSVKWWHIFTRDQCDAINRLVCYFTLPLFTIEFTAHVDPFNMNYRFIAADVLSKVIIVTVLALWAKYSNKGSYCWSITSFSLCTLTNSLVVGVPLAKAMYGQQAVDLVVQSSVFQAIVWLTLLLFVLEFRKAGFSSNNISDVQVDNINIESGKRETVVVGEKSFLEVMSLVWLKLATNPNCYSCILGIAWAFISNRWHLELPGILEGSILIMSKAGTGTAMFNMGIFMALQEKLIVCGTSLTVMGMVLKFIAGPAAMAIGSIVLGLHGDVLRVAIIQAALPQSITSFIFAKEYGLHADVLSTAVIFGMLVSLPVLVAYYAALEFIH

>AtPIN6

MITGNEFYTVMCAMAPLYFAMFVAYGSVKWCKIFTPAQCSGINRFVSVFAVPVLSFHFISQNNPYKMDTMFILADTLSKIFVFVLLSLWAVFFKAGGLDWLITLFSIATLPNTLVMGIPLLQAMYGDYTQTLMVQLVVLQCIIWYTLLLFLFELRAARLLIRAEFPGQAAGSIAKIQVDDDVISLDGMDPLRTETETDVNGRIRLRIRRSVSSVPDSVMSSSLCLTPRASNLSNAEIFSVNTPNNRFFHGGGGSGTLQFYNGSNEIMFCNGDLGGFGFTRPGLGASPRRLSGYASSDAYSLQPTPRASNFNELDVNGNGTPVWMKSPAAGRIYRQSSPKMMWESGQRHAAKDINGSVPEKEISFRDALKAAPQATAAGGGASMEEGAAGKDTTPVAAIGKQEMPSAIVMMRLILTVVGRKLSRNPNTYSSLLGLVWSLISFKWNIPMPNIVDFSIKIISDAGLGMAMFSLGLFMALQPKMIPCGAKKATMGMLIRFISGPLFMAGASLLVGLRGSRLHAAIVQAALPQGIVPFVFAREYNLHPDLLSTLVIFGMIVSLPVTILYYVLLGL

>AtPIN7

MITWHDLYTVLTAVIPLYVAMILAYGSVRWWKIFSPDQCSGINRFVAIFAVPLLSFHFISSNNPYAMNLRFIAADTLQKLIMLTLLIIWANFTRSGSLEWSITIFSLSTLPNTLVMGIPLLIAMYGEYSGSLMVQIVVLQCIIWYTLLLFLFEYRGAKILIMEQFPETGASIVSFKVESDVVSLDGHDFLETDAQIGDDGKLHVTVRKSNASRRSFYGGGGTNMTPRPSNLTGAEIYSLNTTPRGSNFNHSDFYSMMGFPGGRLSNFGPADMYSVQSSRGPTPRPSNFEESCAMASSPRFGYYPGGAPGSYPAPNPEFSTGNKTGSKAPKENHHHVGKSNSNDAKELHMFVWGSNGSPVSDRAGLQVDNGANEQVGKSDQGGAKEIRMLISDHTQNGENKAGPMNGDYGGEEESERVKEVPNGLHKLRCNSTAELNPKEAIETGETVPVKHMPPASVMTRLILIMVWRKLIRNPNTYSSLIGLIWALVAFRWDVAMPKIIQQSISILSDAGLGMAMFSLGLFMALQPKLIACGNSTATFAMAVRFFTGPAVMAVAAMAIGLRGDLLRVAIVQAALPQGIVPFVFAKEYNVHPAILSTGVIFGMLIALPITLVYYILLGL

>AtPIN8

MISWLDIYHVVSATVPLYVSMTLGFLSARHLKLFSPEQCAGINKFVAKFSIPLLSFQIISENNPFKMSPKLILSDILQKFLVVVVLAMVLRFWHPTGGRGGKLGWVITGLSISVLPNTLILGMPILSAIYGDEAASILEQIVVLQSLIWYTILLFLFELNAARALPSSGASLEHTGNDQEEANIEDEPKEEEDEEEVAIVRTRSVGTMKILLKAWRKLIINPNTYATLIGIIWATLHFRLGWNLPEMIDKSIHLLSDGGLGMAMFSLGLFMASQSSIIACGTKMAIITMLLKFVLGPALMIASAYCIRLKSTLFKVAILQAALPQGVVPFVFAKEYNLHPEIISTGVIFGMLIALPTTLAYYFLLDL

>CvPIN

MWEFQDVLNQDLAILLVMAAGALSVWRTLVFRQDDMRIFNVFLFKFTLPASVILGLGLKTDLYKADIWRFVGAFLMMRGIMLAACALVFGGLLRRSLGEVTANWLSTTWISTVILGTPLLRALLGPQYANLGVVAGISSFIFQLPLMLILFEVHTWRQQTIHGALPEQQQAPPQPQGQDSAAERRRGGDDGSQRAGDGSKRLAAAKQQPTVLSVVDASSVPPPRGAAAAGRSSLGDMMRQRLLGCMGWHMTRKQGQRLGLRLVMNHVLWGVGLGLVLSLSTLGPRYLNPGTLPAQPNCTYAVGAGFIFLLLEYFARCTEPVAFFATGKQPVRMWMVRPRPIATGWLRAAAYMAVKLVLVPALMVGCCFAVGLEGANARAAVLVATLPVSAAAFALSKTYSVGEDVAVTNVFLGNLLVLPTTIAWLEFMDGIGLFMVAPKATAPAPTCVAWDA

>CsPINa

MRVFNIFLFRFTLPMSVILGLGIKTDIYDPDVWRFVGAFLMMRAIMLLACVVVVGGLQRGTLGDVTVNWLSTTWISTVILGVPLLKALLGPQYSSLGVVAGISSFIFQLPLMLILFEVHVWRQEVLHGTLPETQPNPATNGGSAPLLTDGFGEHGKESRPDSPDGKVVSSADVHPEPAAARSQRAVLSAADASALPPPDKTARSKSSLRRMGDNLIGCLHFRMTKSQGKRLGLRLVMNHVLWGVGIGLILSLSKIGPKWLDPGPSPPCKVNCSYAVGAGFLYILCDYFARCTEPVAFFATGMWMVRPKPIACGYIKAAIYMLIKLVLVPALMVGCCFTVGLEGAIARAAVLVATLPVSAAAFALSKTYNVGEDIAVANVFLGNLLVLPTTIIWLEFMDGVNLFPAPPAKVPSTC

>CsPINb

MRVFNIFLFRFTLPMSVILGLGIKTDIYDPDVWRFVGAFLMMRAIMLLACVVVVGGLQRGTLGDVTVNWLSTTWISTVILGVPLLKALLGPQYSSLGVVAGISSFIFQLPLMLILFELPLMLILFELPLMLILFEVHVWRQEVLHGTLPETQPNPATNGGSAPLLTDGFGEHGKESRPDSPDGKVVSSADVHPEPAAARSQRAVLSAADASALPPPDKTARSKSSLRRMGDNLIGCLHFRMTKSQGKRLGLRLVMNHVLWGVGIGLILSLSKIGPKWLDPGPSPPCKVNCSYAVGAGFLYILCDYFARCTEPVAFFATGMWMVRPKPIACGYIKAAIYMLIKLVLVPALMVGCCFTVGLEGAIARAAVLVATLPVSAAAFALSKTYNVGEDIAVANVFLGNLLVLPTTIIWLEFMDGVNLFPAPPAKVPSTC

>FsPIN Fistulifera

MVYKYISVVNVGIQIVGTVVLGALSSWIGVFDNTFVVDATKFVFWVALPSLVINGIGVGIDFYSDAFVWDFIKVFLLIRVASLLLSFFLVIAVFRERKGLGDVAVQFLNFSWISTIVHGIPILTAILPDPAMAVFFGLMAGISSFLFQLPLQLFFLEASVLERQMLSPEVTDEEAPTAEKESTEPHEINKVDEDTHLEEEHAEIKLESDSKFETPKSRLALWLELVSRRDILAQVTGRVLRNPVLWGICIGFIISLSTFGKTYLRPLNDEKEPNTNYVEGLEWFVALLNWLGDMVSPLSLFSMGVWMHQQGKDLISISWLELAGSMLVKLVLIPLLMVGLVDVYDLPNTYARSAVLVAALPISLAAFSLGKQFDIGEASLAANVTMGTILMLPTLLAWIEVMDHFELYLI

>SrPIN Salpingoeca rosetta (strain ATCC 50818 / BSB-021)

MTQVVDVVEAAVPLLGLMLTGYCFARFRRKAFPPQSVFLLNSFVFLLGIPSLVFKALATKDLDKLSWRFILGYMLLRTVFGLLSAVPLLWQKTNDKIGDFLINLISSTWINTVIFGIPMLESLYGPRVKILNVLAAISSVIYQLPIMLMFFEYREVLKNGSGNGSRSGGAAELRSVRASAAKTQDEALAMNQVSISSLNRQEVPQQVVPPDTRDAHVVHEALQYQQHQQHHQHQQHHQQQQPASADEEVTMYTAANLPHTSSYTAAISNTNSGRNSSDDSNNSSSSSNSNNSRGALVAAATIAPVDGNEAEDDTMPRTFFGVLWIVIKRVIPKLLRNHPFVGICLGLLWSLILRMAADENNFPKVIDTFVQYFADCVTPVASFCTGMFMCGHERDVIRMWKPLLVFLLAKMIAMPALAMLVCVIVDLDGIEARSAVLLASLPVALASFTLITTYMKERPSAMALTSSLIIVGSILMPFVFAAWNELLIAGDVFGDIPDGTTYTG

>TfPIN Tritrichomonas foetus

MSDYLATVNAGVSVLLIIIVGYVCTWLKIVPAKDFGTLNLFTAKCCFFFMTFRSLAGKDKDVLDFRPLAISVLMSLTLYLIFAIIIFLPFNDSFGLYISIVFPTVYVNYFISGLPIFLALWDESETAVITVILIANDLMCSPIFLTLAKIHELRIQRQKRKTEQMEMKSIDDNIEGDSYSTTSSELESSSISQKQTTEKNNTSNFNNCKNNTTIHQNHTALSETHKNHDQNVNITESDSIEEESAEINIDKKKKKDKINNNDDDDDHYIKKLLLEILNRLIHNMFLVGIVFGMIYLAITSKTCTFLKELMNMLGDCVLPFALFNVGAFLSTQSLIACHWAEALVSLIGRLILGPFLAGIYCYALGFPGRLSQQCIILASMPTAATCFTMTDSAGIGSGVSSTMIMWSAVFMVPVTVFWMWILEVTKLFEE

>GpPIN

MSDPQAAAAKAIAEVGVPIIAIVLLAAMMAAGGAFPTKQALPWLNKFVFYVAIPSTVFKGLATQDFIRDKTFDWGFIGVFLMLRIFVGLLSLIFSVTFGAFGSSDYAKKHKRDWIGHFLTHWIAETYINTLIFGIPVLKSIYGPNALLWNVLASISSLFFQLPIMLVFFEYRKRALEERPSLVAKVVADETDAGSSADPAPADITAIAPPPLNTDKSKDTVVPDTPSVDENDNLGDVFLDGLTWHEKSFTVLKLGLKGIATNPPMWGIFGGIFYSLVFTSTINGAVYTIDSCTGKHLYPIHFDYALQWLSDTITPLASFCIGLFAFAHWRSVAKNWKRETIYILLKMIVVPLFTIPLVLMFNVQPAGKARGAILIAAMPIALASFSLTNQYDVGQDSMASQVIIGTILMTPCCVAWDAIMQSLNLFGNDTLRLK

>KfPIN

MASGGHGSITTGSVYRILSAVVPLYVAICAGYFSVKFKLFTPADIAGINRFVVLIAIPVLSFRFIAGIDLYTISYRLILADTLFKLLVLVVLAVYGLARRQVADLDWLITLWMIATLSNTLIVGVPTLVAMYGPMATDLIGQVVVGQAVIWYPALMILYEIRASRKEERQQVAEEGAELEAGAEVKVADNEGQGSGEARAADTSLDNDEVKLAGEQAAGARCKKLSRVESHPCTCELQRVSTEEERSWPSVDQSQQNPAEEFRIRIKGDTASDGPTQTSPSSSPNHHQPTPPIEASTSKLELQHLTSLERPLHQGEPTNSPTRQPSAVLRGQLSLVVRNRPGVLERKASRAEQVAQWKRTLRILGWKLRKSFTVHAAILGLVYSLVAYKSGFGLPLIVRKSLDVLADTGIGMTMFSLGMFMGSTAIFPCGFCLSLMVHGLHFIIGPAIMGIISFVVGLRGNILRVAIIQAALPQAIVSFSFAKEYDAYPEVLSTSITFGTLMSFPVALAYYVALEHF

>AmTr_v1.0_scaffold00010.98

MISLLDLYHVLTAVVPLYVAMILAYGSVKWWKIFTPDQCSGINRFVALFAVPLLSFHFIAANDPYSMNFRFIAADTLQKLIVLGVLAVWCRVSKRGTLEWTITLFSLSTLPNTLVMGIPLLKGMYGDFSGSLMVQIVVLQCIIWYTLMLFLFEYRGAKLLITEQFPDTAAAIKSFTVDSDVLSLDGREPLETNAEIGEDGKLHVTVRKSNASRSDIFSRRSQGITPRPSNLTNAEIYSLQSSRNPTPRGSSFNHTDFYSMIAGRGNPNPNMSPRHSNFGANDLYSSRGPTPRPSNFEDETKDSDYHHKSRFARGSSENHPLPIAPPNPSIAQYPAPNPAMFSPNSGTKPAVKGANGQQNEGAKDLHMFVWSSSASPVSEVFGQHQEFGASKEVRVGVSPTNEHPPSKGESYPRESQEYERGDFSFGNAGAEGEGQNQGKFGSSSAMELHSRPTDGKATSMPPASVMTRLILIMVWRKLIRNPNTYSSLIGLTWSLVSFRWHVKMPAIVAQSISILSDAGLGMAMFSLGLFMALQPRIIACGNSIAAFAMGVRFLSGPAVMAAASIAVGLRGVLLHVAIVQAALPQGIVPFVFAKEYNVHPDILSTGVIFGMLIALPITLVYYILLGL*

>AmTr_v1.0_scaffold00013.199

MIGLRDVYLVLSAMVPLYIPLGLGYGSVRWWGVLNPEHCNGINRVVALFTLPLFTFNFTASIDPFTMNFRFLGADALSKVFVVAAVALWAKTRGTCCKGPACSWAITGFSLSTLTNTLVVGVPLLKAMYGGIAQDLVVQVSVIQAIVWLSILLFVLECRSSCINGGVVVASETVGNEGSHVAVDVAGTDTGVKVVQVHKRPSLGSLMKVVWRKLALNPNSYASIVGVIWAFISSRWHFSMPLLMENSVSMMAKAGSGMAMFSMGLFMASQEKLIGCGPSLTVLSMVLRFLAGPAAMAVASIAVGLHGDVLHIAIIQAALPQSITSFIYAREYGLHAEVLSTAVIFGMLVSLPVLIAYYVVLELLR

>AmTr_v1.0_scaffold00013.203

MLLAYASVKWWKIFGPDQCAGINRFVAIFAVPLLSFEFISRIDPYNMDLLFVAADSLSKILILSFLASWARLSSKGSIDWVITLFSLATLPNTLVMGIPLVKAMYGDDKESLMIQAVVLQCIIWYTMLLFLFEYRAARLAVMKGFNTGSESGRRDKVEEIESGMREKLEESQKGEDEVVHVMVAKNSNSPSHSSNRVAPHLASTDDDGKELHMFVWPCGCCNSEAVCHQSIQVCSKEEKRKKEEGLVSVQEASMSVEMVKMIMKMVWFKLLANPNTYASLLGLAWALVSCKWNVKKPLILENSVTILSNAGLGMAMFSLGLFMALQPRIIACGTRLAAYGMLIRFFLGPAIMAVASTGVGLRGPILRVAIVQAALPQGIVPFVFAREYNLHPDVLSTAVIFGMIVSLPITVLYYVFLGL

>AmTr_v1.0_scaffold00019.408

MISGKDFYDVLAAMVPLYVAMMLAYGSVRWWKIFTPDQCSGINRFVAVFAVPLLSFHFISTNDPYSMNYRFIAADTLQKVVILIALGAWVNLAKNGSLEWMITLFSLSSLPNTLVMGIPLLKAMYGEFSGALMVQVVVLQSVIWYTLMLFMFEYRGAKVLITEQFPDTAQSITSFKVESDILSLDGREPLLADAEIGQDGKLHVVVRKSLSSRSQISSYKSQCLNSATSVTPRTSNLTGVEIYSLQSSREPTPRGSSFNHTDFYAMFSKLPAGGGQANYGEVYSLNSSRGPTPRTSNFDEEVSTRLGRMRSQDMKDGKSKSGEFGYPAPNPLLLKSPNGDNKRRCSEVGAKELHMYVWSSSASPGSEAHVHGSVARGGSHDFGGAMGLDSKGVNGFTQSPRTGYKGMQDQGENYSPNRKIAPDGDSFVPRDPLEIQSGCISPNKLSAVASPFAQKTPDFNGTTKTRISANNNAQMPPASVMTRLILIMVWRKLIRNPNTYSSLFGLAWSLVSFRWQIQMPTIVHGSIAILSDAGLGMAMFSLGLFMALQPKIIACGTSVAGYSMAVRFLVGPAVMAATSSAIGLRGVLLHVAIVQAALPQGIVPFVFAKEYSVHADVLSTA*

>AmTr_v1.0_scaffold00024.330

MITWKDLYKVLTAVVPLYVAMILAYGSVRWWKIFSPDQCSGINRFVAIFAVPLLSFHFISTNNPYAMNLRFIAADTLQKIIMLFVLALWTNLTRAGCLEWMITIFSLSTLPNTLVMGIPLLIAMYDEYSGSLMVQVVVLQCIIWYTLLLFLFEYRGAKLLIMEQFPDTAGSIVSLKVDSDVMSLDGQEFLQADAEVGDDGKLHVTVRKSTSSSRRSQGLGSLPSLTPRPSNLTGAEIYSLNSSRNPTPRGSNFNNSDFYSMMAGPRHSNFAQPDLYSLQSSRGPTPRPSNFDYPYNSTTATQKNQGGGVHFGIENGNGGNGSSGYPAPNPEVFASSNVNSKPLHSRRGGSDQLLQQPRSPQRSANNNRESHSQSNHHDAKELHMFVWSSSASPVSEGGGLHVFGGPDFARNEQPGRGSDHGAKEIRMLVSDHLPNGETKAIPQSGDDYEGEDFSFSNRVMGEMGEESLPLEKEGPNLSKFGSSSTVELHPKPGPEDWRKKQMPPASVMTRLILIMVWRKLIRNPNTYSSLIGLIWSLIAFRWDVAMPKIVKKSISILSDAGLGMAMFSLGLFMALQPKIIACGNSVAAFAMAVRFLTGPAVMAAASIAVGLRGKLLHVAIVQAALPQGIVPFVFAKEYNVHPAILSTGVIFGMLIALPITLVYYIVLGL*

>AmTr_v1.0_scaffold00038.32

MITASAFYDVFTAMVPLYVAMFLAYGSVKWWRIFTTDQCSVINRFVAIFAIPFLSLQIISSINPYQMNLKFILADTLQKLLILFVLVLWGSFSSWGSLDWVITLFSISTLPNTLIIGIPLLRAMYGVEINGLLVQIVVLQGLVWYTLLLFLFELKAKIEMGPQIPVSMGSQVGSDVSPVHAQSSVVSCKGDLEGDGEMHSKEIEDEEAPSKDSEDEGEATCSSVKMIHLFFLVLSKVIKNPNTYASVLGLVWGLIACRWNIHMPKIVQNSISILADAGLGMAMFSLGLFMASQNKIIACGKRRATLAMVVRFLLGPAVMAACSLAVGLRGKLLHVAIVQAALSQAITPFVYAKEYNVYPDILSTMVVFGMLVSLPITLAYYVLLGI*

>AmTr_v1.0_scaffold00038.35

MAGFYEILSTMVPLYLAMLLAFASVKWWRMFSDEQCSGINKFVANFAAPLLSFKIIYSNDPYKMNHKLILADTMQKLLILFALMLWVKLTKRGSFDWMITLFSMSTLPNTVILGIPLLRAMYGEESEQILFQIVVLQFVVWYPFLLFLYELREAKGAVGRRVSTAVINGSTVVLEVEARSETRMSSLSLATVDGQGSTTAMNGSVVLVVEASAKENSLGYLNGEEGTANSKEREVQVEAAPPQMRMINLLILVWSKVIKNPNTYSSILGLVWGLISFRFNIRMPKIVDKSVTILSVTGLGLSMFSLGLFMASQPNLMPCGKRNATIAMFVRFSFSVVVMSMCSILLGLQGNLLKVAIVQFCNPSYASYAVFRLVGSTGKK

>AmTr_v1.0_scaffold00038.38

MISLADLYAVFTAMVPLYVAMFLAYASVKRWEIFTPEQCSGINRFVATFAIPFLAFNIISSTDPYKINLKFILADTMQKLLILFVLILWAKFTRWGSFNWMITLFSLSIMPNTLIIGIPLLRPMYGEECIGLLVQVVVLQLVVWCPIFLCLYELHAAKVTMNGKVRFTNDTNEGKEGDEMMNVEVIFKKKNTPADECPGDLAHSNEREVQGEVTHPRMTTTNLFLSVLSKVMKNPNTYGSVLGLIWGLISFRWNISMPKIIHKSVSILSVTGLGMAMFSLGLFMGSQRKIIACGKRKVILSMVVRFIFSSTVMAMCSIAIGIRGIALKTAIIQAALPQGVITFVYAKEYQVHEEVLSTA

>AmTr_v1.0_scaffold00101.123

MIKGSDLYHVLTAVVPLYVAMILAYGSVKWWKIFSPDQCSGINRFVALFAVPLLSFHFISTNNPYAMNLRFIAADTLQKLIVLSVLFFWSKTSRRGCLEWAITLFSLSTLPNTLVMGIPLLKGMYGDFSGSLMVQIVVLQCIIWYTLMLFLFEYRGARLLISEQFPDNAGSIISVAVESDVLSFDGREPLQTEAEIGEDGKLHVRVRKSTSSRSEIFSRRSQGLNSGNSMTPRPSNLTNAEIYSLQSSRNPTPRGSSFNHSDFFSMVQGKNAGVSPRQSNFGNSGVEEERERANGVYHNLHNQKQGKQGRKENEGRDLHMFVWSSSASPVSEGGIHVFRGGEFGHENHGGADLRLGMGPHGGEVAKEANHESYDEFGRDDFSFGNRPLSQGDESLPRDGHGPSLSKFGSSSTAELHPKASGDGGPKPSAMPPTSVMTRLILIMVWRKLIRNPNTYSSLIGLTWSLVCFRWGIQMPVIVARSISILSDAGLGMAMFSLGLFMALQPRIIACGNSIAAFAMAVRFLTGPAIMAVASIAVGLRGVLLHVAIVQGHLWHADCFTHHPGLLHFAWTMMTTSKMAAKPAQRLGGLAFSLKNLERREREKLYLFAEN*

>Bradi1g31530.1.p

MITGRDIYDVLAAVVPLYVAMFMAYGSVRWWGIFTPDQCSGINRFVAVFAVPLLSFHFISTNDPYAMDYRFLAADSLQKLVILAALALWHNLLSRFKPNNGGPAQLDWTITLFSLATLPNTLVMGIPLLRAMYGDFSGSLMVQIVVLQSVIWYTLMLFLFEYRGAKALISEQFPPDVGASIASFRVDSDVVSLNGREPLQADAEVGTDGRVHVVIRRSASGSTTGAGHGGGGYGIGGHGRASRGMTPRASNLTGVEIYSLQTSREPTPRQSSFNQNDFYSMFSAAGTGTGTSKLASPSPMGQQGQNNGNNGGGARVGLDEQVANKFKGGVDYPAPNPGMMAAPAPRKKELGGSNSNSNKELHMFVWSSSASPVSEANLRNAVISHAASTDFAAAPPAAAASATPHDAAATPKGVSGTVTPVMKMKDGGAVELEIEDGTMKSPAAGLAAKFPVTGSPYVAPRKKGAGGGLVEEEAAHPMPPASVMTRLILIMVWRKLIRNPNTYSSLIGLVWSLVSFRWNIQMPTIIKGSISILSDAGLGMAMFSLGLFMALQPKIISCGKSVATFAMAVRFLTGPAVIAATSIAVGLRGVLLHVAIVQAALPQGIVPFVFAKEYNCHPQILSTAVIFGMLIALPITILYYILLGI

>Bradi1g45020.1.p

MITGTDFYHVMTAMVPLYVAMMLAYGSVKWWRIFTPDQCSGINRFVALFAVPLLSFHFISTNNPYAMNLRFIAADTLQKLIVLALLTLWSHLSRNGSLEWTITLFSLSTLPNTLVMGIPLLKGMYGDESGSLMVQIVVLQCIIWYTLMLFMFEYRGARILITEQFPDTAGAIASIVVDPDVVSLDGRRDMIETEAEVKEDGKIHVTVRRSNASRSDIYSRRSMGFSSTTPRPSNLTNAEIYSLQSSRNPTPRGSSFNHTDFYSMVGRSSNFAAGDVFGGPGPVVRTGATPRPSNYEEDKAAAAGGNKYPAPNPAMAAAPPKPGKKQQQQGKGEDGKDLHMFVWSSSASPVSDVFGNGAEYNVDAAAVKDVRVAVASPRKGDGRKEKGEDYVERDEFSFGNKGMAGERDAEAGDEKDQGNAGGVVAATAMPPTSVMTRLILIMVWRKLIRNPNTYSSLIGLVWSLVCFRWNFEMPAIIMKSIAILSDAGLGMAMFSLGLFMALQPRIIACGNKRASFAMAVRFLTGPAVMAAASIAVGLRGKLLHVAIVQAALPQGIVPFVFAKEYSVHPDILSTAVIFGMLIALPITLVYYILLGL

>Bradi2g15610.1.p

MISWHDLYTVLSAVVPLYVAMALAYGSVRWWGVITPDQCGGINRFVAVFAVPLLSFKVISGTDLYAMDLRFAAADTLHKCAVLAALAASARFFPGSGGSRLDWPVTFFSFATLPNTLIMGIPLLVAMYGSHAGDLMVQLVVLQCIVWYTLLLFLFELRAARALISAAAAAAGAGGRRERVESFVVDPDVVSLDGSHAEAQAEVAPDGSMRLTVRRASASASASRRSLMAGGVGAATPRASNLTGVEIYSACSSPRVSLGAHADLTAHPAAPASASASALRASSFGAADLFSLHSSRQHTPRPSASASFDERNPRATAAVAPVNDDDGAGDDDRKNSDARVLDWSSGASEVSGLPVFRAGVGRDSGRLRLQPIGSGSSRMIRAARGPAPTCAKPEAAVHEAVAKLEAGLAKTTTDERQKHEQQGVAKDGGAGQKMPPAGVMTRLILTMVWRRLIRNPNTYASVVGLTWSLVEFRYKIAMPAIVANSISILSNTGLGMAMFSLGLFMAMQPKLIACGHTVAATTLAVRFLFAPATMAAAAATVGLRGTLLRVAIVQAALPQGIVPFVFAKEYNLHAAALCTGVIFGMLIALPIALVYYIILGLL

>Bradi2g44987.1.p

MISSHDLYTVLAAVVPLYVAMILAYGSVRWWGIFTPDQCSGINRFVAIFAVPLLSFHFISASDPYQMNLRFLAADTLQKLLVLAALAACSRLIPSLGLDWSVTLFSLSTLPNTLVMGIPLLVAMYGPDAGSLMVQIVVLQCIIWYTLLLFLFEFRAARALIADQFPDTAASIASLHVDPDVVSLDGGRAETEAEVAPDGRLRVTVRRSSVSRQSLSLLGVTPRPSNLTGAEIYSVNSSQNPTPRGSNFNHADFFATVGAPPPPTTPAGARGSSFGTSSEHFSLHTSSQGPTPRGSNFDEHSAGAGGRKPAAAGAHSSHDAKELHMFVWSSSTSPVSEVSRLPADRARLDAKEIRMVVPADPPQNGSCKEAAVEDDGEAAEAGRERLTKLSSSSTAELRPNVYADTAGAEDAKRPRQQHQMPPASVMTRLILIMVWRKLIRNPNTYSSLIGLVWSLAAFRWHISMPSIVGKSISILSDAGLGMAMFSLGLFMALQPSIIACGNTVAVVSMVVRFIVGPAVIAAASAAVGLRGTLFKISIVQAALPQGIVPFVFAKEYNVHPTILSTAVIFGMLVALPITLAYYAILGLVTV

>Bradi2g48170.1.p

MISWIAIYHVLEATVPLYVAMILAYLSIKWWKLFTPEQCSGINKFVAKFSIPLLSFQVISTNNPYDMNLKLILADILQKLLALLGFAAISRACCAEKFDWLITGFSLSTLPNTLIVGIPLLKGMYGDEALNLISQIVVLQSLIWYTLLLFLLEFRAAKGIVPTTSSEITAEEEPGTLGTTQQRYQEGQAKGVSAKCSSAFNFLLVVGKKLVMNPNMYACLIGLIWALVSFRWQIQLPVIISNSIRILSDGGLGMAMFSLGLFTALQTKIIVCGTKRMLLSLGIRFFLGPALMVISSYAVGMRGILLKVAIVQAALPQGIVPFVFAKEYNLHADILSTAIIVGMMVAVPAALAYYFVLDQPRF

>Bradi2g52640.1.p

MITGSAVYHVVEAMAPLYTAALLGYASVRWLGAFSAEQCAGINHFVAIYAVPVLIFHMVSTNDPYAMSGRLIAADTLQKAAMLLALVAWAAWPARWRRRGNNNKAVAAAAAAVSPLQWVVTAFSVAALPNTIIMGVPLLGGMYGDVSKGLMKQIVVMQFCVWYNVVIFIYEYMAARRAATVDGTARIRDANDDDIVVVAAERAHEVTVKIEITEVAAAPPVPQEGVAGETKTTTVAKETSTAETEGVSPPKKTAPSARHVALMAGKKVLKIPNTYASFLGLIWALIAFKCGIKMPKIIDDSLFTIHTTAVGLSMFASGTFIARQSRFVPCGYAVASISMVLKFLIGPVVMLLASLAIGLHGTLLHIAVVQAALPLAVTSFVYAEEYKVHADIMSTGVILGIFISLPVTIVYYILLGL

>Bradi2g58917.1.p

MIGWGDVYKVVAATAPLYFALLLGYGSVRWWRIFTREQCDAVNRLVAFFALPFFTFEFTLHTDPFQVNYRAVAADVVSKAVIVAAIAVWARFHRGVDGAGAWAITSFSLSTLTNSLVVGVPMARAMYGEWAQQLVVQLSVFQAIVWLTLLLFVLEVRKAAIGMYIVPAESASSPEAVIKGGGVDVEAANGEAAAAVVGVGRPTVWRLFKTVAHKLALNPNTYASFVGITWACVANRLHIELPSVLEGSVLIMSKSGTGMAMFSMGLFMAQQEKILACGPSLAALGLALKFALGPAAMAVGSIAVGLRGDVLRVAIIQAALPQSITSFIFAKEYGLHADVLSTA

>Bradi3g41080.1.p

MIAWGDIRKVVSAMAPLYFALGLGYGSSRWWHLLASPEQCAAVNTLVAFFSMPFFTFDFVSGVDPSAVNFRVLAADALAKLLAILAAWAWAWARQYDDWSWPITGFSLATLSNTLVVGVPLLEAMYGDWARELMVQVAVAQSVVWVPLLLLAFELRNACCVLHLQPEPETKTKDGGGDVELAAAAAARVERDPNARAGNGNGNKKIRRCWAMARTVGIKVASNPNVYASVLGVAWACAAYRWRVGMPGVVTGALQVMSRTGTGMSMFSMGLSMAQQERMVACGAGLAALGMALRFVAGPAAALAGAAAMGIRGNVLRFVVVQAALPQSIASFVFAKEYGLHADVLSTAVIFGTLLSLPVLIAYYALLDII

>Bradi3g59520.1

MITAADFYHVMTAMVPLYVAMILAYGSVKWWRIFTPDQCSGINRFVALFAVPLLSFHFISTNNPYTMNLRFIAADTLQKLMVLAMLTAWSHLSRRGSLEWTITLFSLSTLPNTLVMGIPLLKGMYGEFSGSLMVQIVVLQCIIWYTLMLFMFEYRGARMLITEQFPDTAANIASIVVDPDVVSLDGRRDAIETETEVKEDGRIHVTVRRSNASRSDIYSRRSMGFSSTTPRPSNLTNAEIYSLQSSRNPTPRGSSFNHTDFYSMVGRSSNFGAADAFGVRTGATPRPSNYEDDASKPKYPLPASNAAPMAGHYPAPNPAVSSAPKGAKKAATNGQAKGEDLHMFVWSSSASPVSDVFGGGAPDYNDAAAVKSPRKMDGAKDREDYVERDDFSFGNRGVMDRDAEAGDEKAAAAAGADPSKAMAAPTAMPPTSVMTRLILIMVWRKLIRNPNTYSSLIGLIWSLVCFRWNFEMPAIVLKSISILSDAGLGMAMFSLGLFMALQPHIIACGNKVATYAMAVRFLAGPAVMAAASFAVGLRGTLLHVAIVQAALPQGIVPFVFAKEYSVHPSILSTAVIFGMLIALPITLVYYILLGL*

>LOC_Os02g50960.1

MITAADFYHVMTAMVPLYVAMILAYGSVKWWRIFTPDQCSGINRFVALFAVPLLSFHFISTNNPYTMNLRFIAADTLQKLMVLAMLTAWSHLSRRGSLEWTITLFSLSTLPNTLVMGIPLLKGMYGEFSGSLMVQIVVLQCIIWYTLMLFMFEYRGARMLITEQFPDTAANIASIVVDPDVVSLDGRRDAIETETEVKEDGRIHVTVRRSNASRSDIYSRRSMGFSSTTPRPSNLTNAEIYSLQSSRNPTPRGSSFNHTDFYSMVGRSSNFGAADAFGVRTGATPRPSNYEDDASKPKYPLPASNAAPMAGHYPAPNPAVSSAPKGAKKAATNGQAKGEDLHMFVWSSSASPVSDVFGGGAPDYNDAAAVKSPRKMDGAKDREDYVERDDFSFGNRGVMDRDAEAGDEKAAAAAGADPSKAMAAPTAMPPTSVMTRLILIMVWRKLIRNPNTYSSLIGLIWSLVCFRWNFEMPAIVLKSISILSDAGLGMAMFSLGLFMALQPHIIACGNKVATYAMAVRFLAGPAVMAAASFAVGLRGTLLHVAIVQAALPQGIVPFVFAKEYSVHPSILSTAVIFGMLIALPITLVYYILLGL*

>LOC_Os12g04000.1

MEQFPDTAASIVSFRVDSDVVSLAGGGGGAAELQAEAEVGDDGKMRVTVRKSTSSRSEAACSHGTQSHSQSMQPRVSNLSGVEIYSLQSSRNPTPRGSSFNHAEFFNIVGNGKHGDEEKGAAGGGGHSPQPVVGKRKDLHMFVWSSSASPVSERAAAAAAAGAVHVFGGGGADHGDAKGAQAYDEYSFGNKNEKDGPTLSKLGSNSTAQLRPKDDGEGRAAAMPPASVMTRLILIMVWRKLIRNPNTYSSLLGVIWSLVSYRWGIEMPAIIARSISILSDAGLGMAMFSLGLFMALQPRIIACGNSLASYAMAVRFLVGPAVMAAASIAVGLRGVLLHIAIVQAALPQGIVPFVFAKEYNVHPNILSTAVIFGMLIALPITLVYYILLGL*

>LOC_Os06g44970.1

MITGRDIYDVLAAIVPLYVAMFLAYGSVRWWGIFTPDQCSGINRFVAVFAVPLLSFHFISTNDPYSMNYRFLAADSLQKLVILAALAVWHNLLSRYRRNGGAAASLDWTITLFSLSTLPNTLVMGIPLLRAMYGDFSGSLMVQIVVLQSVIWYTLMLFLFEYRGAKALISEQFPPDVGASIASFRVDSDVVSLNGREALQADAEVGRDGRVHVVIRRSASASTTGGGGGAARSGVSRAYGASNAMTPRASNLTGVEIYSLQTSREPTPRASSFNQADFYAMFSGSKMASQMASPMAQHGGAGGRAQGLDEQVTNKFASGKAADPPSYPAPNPGMMPAPRKKELGGSNSNSNKELHMFVWSSSASPVSEANLRNAVNHAASTDFASAPPPAAVPVGGATPKGVSGSVTPAAKNGGGELEIEDGLKSPAAGLAAKFPVSGSPYVAPRKKGGGADVPGLAEAAHPMPPTSVMTRLILIMVWRKLIRNPNTYSSLIGLVWSLVSFRWNIQMPSIIKGSISILSDAGLGMAMFSLGLFMALQPKIISCGKTVATFAMAVRFLTGPAVIAATSIAIGLRGVLLHVAIVQAALPQGIVPFVFAKEYNCHPQILSTAVIFGMLIALPITILYYVLLGI*

>LOC_Os01g69070.1

MIGWGDVYKVVAATVPLYFALFLGYGSVRWWRIFTREQCDAVNRLVAFFALPFFTFEFTLHTDPFQVNYRAVAADVISKAVIVAVIGAWARFMSKGGCAVSWSITSFSLSTLTNSLVVGVPMARAMYGEWAQQLVVQLSVFQAIVWLTLLLFVLEVRKAAIGMYVDGAEAAAAAGKDVEAAGAAAAAGTVVVAAAAGKPSLWALVKVVAHKLARNPNTYASFVGITWACLANRLHIALPSAFEGSVLIMSKSGTGMAMFSMGLFMAQQEKIIACGTSFAALGLVLKFALGPAAMAIGSIAVGLRGDVLRVAIIQAALPQSITSFIFAKEYGLHADVLSTAVIFGMLVSLPLLVGFYIVLELIR*

>LOC_Os08g41720.1

MAPLYFALGLGYGSVRWWRFFTAEQCAAINTMVVYFSMPFFTFDFVVRTDPFAMNYRVIAADAVSKAIAIAAMAAWARTRCGCAAAKAGAQSWSITGFSLAALNNTLVVGVPLLDAMYGRWAQDLVVQIAVVQSMVWFPLLLMAFELRKAWVVGGGGGVGPAVMSSSSPPEKQSDVEMNGAVVAAPGGGGGVRLPFWATARTVGLKLARNPNVYASVLGVVWACIAYRWHLSLPGIVTGSLQVMSRTGTGMSMFSMGLFMGQQERVIACGAGLTALGMALRFVAGPLATLVGAAALGLRGDVLHLAIIQAALPQSIASFVFAKEYGLHADVLSTAVIFGTLISLPILIAYYAVLGFV*

>LOC_Os09g32770.1

MAPLYFALGLGYGSVRWWRLFTADQCDAVNRLVACFAVPFFAFDFAARIDPFALSYRVLAADALSKLAVALALAACAAAASTRCCGSGGGKRGGGGGFSWCITGFSLATLNNTLVVGVPLLDAMYGKWARDLIVQISVVQTIVYFPLLLLAFEVRRATTAAAAPPPPPTGTDDDDVEDGAAAAATAAAARRSLWPLVRAVWLKVARNPNVYAGVLGVAWACVTNRWHVETPSIIEGSVLIMSKTGVGLSMFSMGLFMALQDKIIVCGAGLTVLGMALRFVAGPAATAVGAFALGLRGDLLRLAIIQAYTHLHEIHSPSSFFLLSPEICLNSNGCMHDTSPHDETARDEQAALPQSITTFVFAKEYGLHAEILSTAVIFGTLASLPVLIVYYIVLGFIR*

>LOC_Os01g51780.1

MVSWKDIYLVLEATVPLYVAMILAYLSIKWWKLFTPEQCSGINKFVAKFSIPLLSFQVISSTDPYDMNIKLIYSDILQKSLALLGFAAISKACCAEKFDWLITGFSLSTLPNTLIVGIPLLKGMYGEQAGKLLSQIVVLQSLIWYTLLLFLFELRAANGMATTTSSETTGLIWALVGFRWHIRLPLIVSNSIRMLSDGGLGMAMFSLGLFTALQTKIIACGAKRMLLALAIRFFLGPALMGMSSYAIGMRGVLLKIAIVQAALPQGIVPFVFAKEYNVQADILSTAIIVGMMVAVPVALAYYFAMIIPAIK*

>LOC_Os01g58860.1

MITGSEVYQVVEAMAPLYTAAALGYGSVRWLKAFSNEQCAGINHFVALYAVPVLIFDMVSTNNVYKMNGRLIAADTLQKAVLLLGLMAWALWERSRARGAGAKAKAAVSSPLQWVITCFSVASLPNTIIMGVPLLNGMYGPVSKDLMKQIVVMQFCIWYNVIIFLYEYMAARRSASAPPASSEGSAKISPSSPVKAAAAAADTNGNAVAADRPQEVAVNIEITEMAASTARDGVSGETTAAAKEVSSGEVAPVEEEEASAPAPSMKHVIWMAVKKLLQIPNTYASFLGLIWSLIAFKCGFSMPKIVEDSLFTIRTTAVGLSMFSSGTFIARQSRFVPCGYKIASFSMVIKFLIGPVVMLFASLVIGMHGTLLHIAVVQAALPLAVTSFVYAEEYKVHADIMSTGVILGIFISLPVTIVYYILLGL*

>LOC_Os01g45550.1

MISGHDFYTVMAAVVPLYVAMFLAYGSVRWWGIFTPDQCSGINRFVAIFAVPLLSFHFISTNDPYAMNLRFLAADTLQKLLVLAGLAAWSRLPSRTGAPRLDWSITLFSLSTLPNTLVMGIPLLIAMYGPYSGSLMVQIVVLQCIIWYTLMLFLFEFRAARMLIADQFPDTAASIVSLHVDPDVVSLEGGHAETEAEVAADGRLHVTVRRSSVSRRSLLVTPRPSNLTGAEIYSLSSSRNPTPRGSNFNHADFFAMVGGGPPPPTPAAVRGSSFGASELYSLQSSRGPTPRQSNFDEHSARPPKPPATTTGALNHDAKELHMFVWSSSASPVSEVSGLPVFSGGGGGGALDVGAKEIHMVIPADLPQNNGSGKEHEEYGAVALGGGGGGENFSFGGGKTVDGAEAVDEEAALPDGLTKMGSSSTAELHPKVVDVDGPNAGGGAAGAGQYQMPPASVMTRLILIMVWRKLIRNPNTYSSLLGLAWSLVAFRIVHGAAAQHHRVWQISRRRLHGRPLPRGPCRHGRRVNRHRTPRDAPARRHCSGGSTTRDCAFCFCKRIQCPPGHPEHSGNFWHANSSSNHIAVLHPSWTMIKKAYGRSHIKRKKWGQRERKKSDPVHLKQRMLICIACCRTLPMMTRQRSCYRAIYLCDICTRDNDRMYSNITDIRYVFC*

>LOC_Os05g50140.2

MISWHELYMVLSAVVPLYVAMMVAYGSVRWWGVLTPEQCSGINRFVAVIAVPLLSFHFISSSDPYAMNLRFVAADTLQKVLVLAALAAWSRFPARFVPPAWPPLDCSITLFSVSTLPNTLVMGIPLLVSMYGPYSGDLMVQIVVLQSIVWYTLLLFLFEFRAARVLIAAQFPDTAASIAAVHVDPDVVSLEGSQAEAHAEVAPDGRLRMVVCRSSVSRRSAAAAATPRASNLTGVEIYSISSRNATPRGSTFTLADIPGHQPPNSALRASSFGAADLFSLHSSSRQHTPRPSSFDEHAAARARASATVAPTNDLKDTHMIEWSSGASAASEVTGLPVFRSGRETRRLVPSDAPSIASSRVIRPPPGATGGERAASFNKAVGGQDELAKLEAGAKTEQQTTAVTTTTKGGGAAGAERARGQQNAPAGVMLRLILTTVWRRLIRNPNTYASLIGLTWSLIAFRFHITMPIIVAKSISILSDAGLGMAMFSLGLFMATQPKIIACGYSVAAASMGVRFFFGPAIMAAASAAVGIRGTLLRIAIVQAALPQGIVPFVFAKEYNLHATILCTLVIFGMLIALPITLVYYIILGLL*

>Zm00001d044812_P001

MITGTDFYHVMTAMVPLYVAMILAYGSVRWWRIFTPDQCSGINRFVALFAVPLLSFHFISTNNPYTMNIRFIAADTLQKLIVLALLTAWSYLSRRGCLEWTITLFSLSTLPNTLVMGIPLLKGMYGDFSGSLMVQIVVLQCIIWYTLMLFMFEYRGARILITEQFPDTAGAIASIVVDPDVVSLDGRNDAIETEAEVKEDGKIHVTVRRSNASRSDIYSRRSMGFSSTTPRPSNLTNAEIYSLQSSRNPTPRGSSFNHTDFYSMVGRSSNFAAGDAFGLRTGATPRPSNYEEDPQGKANKYGQYPAPNPAMAAQPAKGLKKAANGQAKGEDGKDLHMFVWSSSASPVSDVFGNGAAEYNDAAAVKEVRMAVASPRKVAADGRKERGEDFAERDDFSFGNRGAAERDAEAGDEKAAAAAAAAQGNAGAVAAPAAMPPTSVMTRLILIMVWRKLIRNPNTYSSLIGLIWSLVCFRWNFEMPAIILKSISILSDAGLGMAMFSLGLFMALQPRIIACGNKVATFAMAVRFLTGPAVMAAASFAVGLRGTLLHVAIVQAALPQGIVPFVFAKEYGVHPDILSTAVIFGMLIALPITLVYYILMGL*

>Z.maysQ0MWY1SV1

MITGTDFYHVMTAVVPLYVAMILAYGSVRWWRIFSPDQCSGINRFVALFAVPLLSFHFISTNNPYTMNLRFIAADTLQKLMVLAMLTAWSHLSRRGSLEWTITLFSLSTLPNTLVMGIPLLKGMYGDFSGSLMVQIVVLQCIIWYTLMLFMFEYRGARMLITEQFPDNAGAIASIVVDPDVVSLDGRRDAIETEAEVKEDGRIHVTVRRSNASRSDIYSRRSMGFSSTTPRPSNLTNAEIYSLQSSRNPTPRGSSFNHNDFYSMVGRSSNFGAADAFGIRTGATPRPSNYEDDASKPKYPLPVVNATSGAGAAHYPAPNPAVAAAPKGARKAATNGQAKGEDLHMFVWSSSASPVSDVFGGGAPDYNEAAAAKSPRKMDGGKDRDDYVERDEFSFGNRGAMDRDAEAGDGKAAAAGGDPSTVAAPTAMPPTSVMTRLILIMVWRKLIRNPNTYSSLIGVIWSLVCFRWNFQMPAIVLQSISILSDAGLGMAMFSLGLFMALQPRIIACGNKVATFAMAVRFLTGPAVMAAASFAVGLRGTLLHVAIVQAALPQGIVPFVFAKEYNVHPDILSTAVIFGMLIALPITLVYYILLGL

>Zm00001d052269_P001

MISAADLYHVMTAVVPLYVAMILAYGSVRWWRIFSPDQCSGINRFVALFAVPLLSFHFISTNNPYTMNLRFIAADTLQKLMVLAMLTAWGHLSRRGRGSLDLDWTITLFSLSTLPNTLVMGIPLLRGMYGDFSGSLMVQIVVLQCIIWYTLMLFMFEYRAARLLIAEQFPGNAAGAIASIAVDPDVVSLVDHGRGDAIETEAEVREDGRIHVTVRRSSASRSDVVYSRRSVGGFSGATPRPSNLTNAEIYSLQSSRNPTPRGSSFNHTDFYSMVGRSSNFGAADAFGAVRTGAGTTPRPSNYEDDASRPKYPLPVVSAAPGVAGHYPAPWSARKAATNGHAKGEDLHMFVWSSSASPVSDFFGGGAPDYNDHAAAAKSPRKMDGGARDRDDYVERDEFSFGNRGAMEDRDAEDPAAAGGDPNAVAAATAMPPTSVMTRLILIMVWRKLIRNPNTYSSLIGVVWSLVCFRWNFQMPDIVLHSISILSDAGLGMAMFSLGLFMALQPRIIACGNKVATFAMAVRFLTGPAVMAAASFAVGLRGTLLHVAIVQAALPQGIVPFVFAKEYSVHPDILSTAVIFGMLIALPITLVYYILLGL

>Zm00001d052442_P001

MITALDLYHVLTAVVPLYVAMTLAYGSVRWWRIFTPDQCSGINRFVALFAVPLLSFHFISTNDPFAMNLRFLAADTLQKVAVLALLALASRGLSSPRALGLDWSITLFSLSTLPNTLVMGIPLLRGMYGASSAGTLMVQVVVLQCIIWYTLMLFLFEYRAARALVLDQFPDGAAASIVSFRVDSDVVSLARGDVELEAEPDGVAGAGAVSSRGGDAGRVRVTVRKSTSSRSEAACSHSHSQTMQPRVSNLSGVEIYSLQSSRNPTPRGSSFNHADFFNIVGAAAKGGGGAAGDEEKGACGGGGGGHSPQPQAVAVPAKRKDLHMLVWSSSASPVSERAAVHVFGAGGADHADVLAKGAQAYDEYGRDDYSSRTKNGSGGADKGGPTLSKLGSNSTAQLYPKDDGEGRAAAVAMPPASVMTRLILIMVWRKLIRNPNTYSSLIGVVWSLVSYRWGIEMPAIIARSISILSDAGLGMAMFSLGLFMALQPRIIACGNKLAAIAMGVRFVAGPAVMAAASIAVGLRGVLLHIAIVQAALPQGIVPFVFAKEYGVHPDILSTAYGPITSHGFITCHS

>Zm00001d042345_P001

MIGWGDVYKVVAATVPLYFALFLGYGSVRWWRIFTREQCDAVNRLVAFFALPFFTFEFTLHTDPFQVNYRAVAADVISKAVIVGVIGCWAGSRVGSAGKGKGAVGWSITGFSLSTLTNSLVVGVPMARAMYGEWAQQLVVQLSVFQAIVWLTLLLFVLETRKAAIGMYVVPGGAADSDGGVPDSPVKDKDVEAAAGDRRPAAGPAAAATVVVVVPVKPSLWALIKVVAHKLSRNPNTYASFVGITWACVANRLHIELPSAFEGSVLIMSKSGTGMAMFSMGLFMAQQEKVLACGPSFAALGLVLKFALGPAAMAIGSIAVGLRGDVLRVAIIQAALPQSITSFIFAKEYGLHADVLSTAVIFGMLVSLPLLVGFYIVLELIR

>Zm00001d031594_P001

MIAWGDIYNVVSGMAPLYFALVLGYGSVRWWRLFTAAQCGAINTLVANFSMPFFTLDFLARADPYAMNYRVLAADAVSKALLAVAAGGGAGSRSSSWAITGFSLAGFNNTLVVGVPLLFAMYGKWAQDLVVQVAVVQALVWFPLLLLGFELRRAWVGGVPRAQGVGVGVGRCSSDDDDGDGHLGGRVGPVSLPPPLKTKDVEMNAAEEEAADDDAPAAAGAIRLWPTVRTVGLKLVGNPMSTPAWSAWCGRASPTGRDHAVRSPCTHHLRCVCTAKCHPAAAVLPSPSRACMRSCADVSRPWRVCACRWDLSLPGIVTGSLGVMSRTGTGMSMFSMGMFMAQQERVIACGAGLAALGLALRFVAGPLATLVGAAAFGLRGDVLRFAIIQAALPQSIASFVYAKEYGVHADVLSTAVILGTLVSLPVLIAYYAVLGIL*

>Zm00001d006082_P001

MIGWGDVYKVVAAMAPLYFALGLGYGSVRWWKLFSPDQCDAVNRLVAYFAVPFFAFDFAARIDPFALSYRVLAADALSKLAVALALAAWATAAASASARRGGGGKGKDRALSWCITGFSLATLNNTLVVGVPLLDAMYGKWARDLIVQISVVQIIVYFPALLLALEARRAGKLAVVAEEAAGDADESGGGGSGGGTTAAQSLWPLVTAVWKKVARNPNVYAGILGVSWACVTNRWHIETPSIIEGSVLVMSKTGVGLSMFSMGLFMALQEKVIVCGAWPTVLGMALRFVAGPAATAAGAVALGLRGDVLRLAVMQAALPQSITTFVFAREYGLHPEVLSTAVIFGTLASLPVLIVYYIVLGLIRC*

>Zm00001d043660_P001

MISWPTIYHVLEETVPLYVAMIVAYLSIQWWKLFTPEQCSGINKFVAKFSIPLLSFQILSTNNPYSMNLKLIFSDVLQKSLSLLGFAVASRVCCEEKFDWLITGFSLSTLPNTLIVGIPLLKGMYGDEAVKLLSQIVALQSLIWYTLLLFLFEFRAARGMAITTSSEAIDEAESGTPGPMQQGHEEGQAKGVSARCYGAFHFLLVVSRKLVTNPNMYASLAGLIWALISFRWRIQLPSIVNNSIRILSDGGLGMAMFSLGVFTALQTKIVACGTKRMLLSLGIRFFLGPGLMVISSYLIGMRGTLLKVAIIQAALPQGIVPFVFAKEYNVHADILSTAIILGMMVAVPVALGYYFVMDHPRL

>Zm00001d043179_P001

MIPGSAVYHVVEAMAPLYTSAVLGYASVRWLKAFSDEQCAGINHFVALYAVPVLIFHMVSTNDPYHMNERLIAADTLQKAVVLLALTAWAFWSHLSRRRGGRGKRQVVQGAESPIKWVVTNFSVASLPNTIIMGVPLLDGMYGPVSGGLMKQIVVMQFCIWYNVVIFLYEFMAARDGSARISPAVPATAGERVVDDENGGSSSVHRHAAADRSHHHHQVVVNIEITEVAPAPAAEAVVASSLMPAAATKEMSPEDDGDANKQVVVAVDVQPPVMHVVWMATKKLLQIPNTYASFLGLIWSLMAFKIGFSMPKIVSDSLFIIYTTAVGLSMFASGTFIARQSRFVPCGYTVASLSMLLKFLIGPVIMLIASLAIGMHGTLLHIAVVQASRPTAPNNAALPLAVTSFVYAEEYKVHADIMSTGSVPFKVILGIFISLPVTIVYYILLGL*

>Zm00001d044083_P005

MISWNDVYTVLTAMVPLYVAMFLAYGSVRWWRIFTADQCSGINRFVAIFAVPLLSFHFISTNDPYAMNLRFLAADTLQKLVVLAGLAACSRLPSRLAAPRLDWSITLFSVSTLPNTLVMGIPLLIAMYGPYAGSLMVQVVVLQCIIWYTLLLFLFEFRAARTLIADQFPDTAAAIASLHVDPDVVSLEGGRAETEAEVAEDGRLHVTVRRSSASRRSLLMATPRPSNLTGAEIYSMSSSRQHSPRGSNFNHADFFAMVDGAPPPPAPGARASSFGAAELYSMHSSRGPTPRQSNFDERSASARSSRPAAVPSHDAKELHMFVWSSGASPVSDVSGLPAFTGGGAGGVNVGAKEIRMVVPAEMPQNGSAGKENENNGAAAAATGEAEGFGFRSGGKTTAEDAEAGEAAGPEQLTKLGSRYSTAELRAKDDGGGAGSAAQQMPPASVMTRLILIMVWRKLIRNPNTYSSLIGLAWSLVAFRWHISMPAVVAKSIAILSDAGLGMAMFSLGLFMALQPKLIACGWRATGVSMAVRFLAGPAVMAAASLAIGLRGTLLRIAIVQAALPQGIVPFVFAKEYNVHPAILSTMVIFGMMIALPITLIYYILLGLKPV

>Zm00001d045219_P001

MISWHDLYTVLCAVVPLYVAMILAYGSVQWWGVLTPDQCSGINRFVAVFAVPLLSFHCISASNPYVMNLRFIAADTLQKVLVLAALAVWSHLPPARGCGGGTKLREPFDWSITLFSLSTLPNTLIMGIPLVVAMYGRYSGDLLVQVVVLQCIVWYTLLLVLYEFRAARVLIAGQCPDGAAARIADVRVDPDVVSLCGSQAEAEAEVAPDGRVRLVVRRSTSASRRSLATPRPSNLAGVEIYSVSSSRNATPRGSSGIAHADAICAAAPPPHGASSLRMSSFGAADLFSLHPTPRPSSFEEQAVRARSAAAVAPSNDPRDAHMFEWSSGASATSEVRGLPVFHGGDIHRGMDPRRLVPSEAPPRAMRPGERVVTGYPADAGQDIVLAKLESGSTEAERKDACGRDGGAGEQQAAAPAGVMMRLIVTMVWRRLIRNPNTYASVVGLTWSLISFRFHFAMPAIVKNSITILSDAGLGMAMFSLGLFMALQPKIIACGNTVAAVTMAIRFFLGPAVMAATSAAVGLRGTLLRVAVVQAALPQGIVPFVFAREYSLHATILSTGVIFGMLIALPIGLVYYVVLGLL

>Mapoly0089s0050.1

MINAHDLYNVLSAVIPLYVAMILAYGSVKWWGILTPDQCGGINRFVAVFAVPLLSFKFIALNDPYTMNYQFIAADAMSKIAILILLGLWARYTKRGSFDWMITIFMLATLPNTLVMGIPLLRAMYGAEAGSLVIQAVVLQCIIWYTMLLFLYEFRAAKTLIMDQFPETAASIVSFKVDSDVTSLDGREPVQTEAEVGDDGKLHVKVRKSVSSSQGISSRRSQGLLSMPSSKALTPRPSNLTGAEIYSLHSSVNLTPRESSFNHTDFYSMISGRGGSPRQSNFTSSDVYSLHSSRGPTPRTSNFNEENSKDLHSYARGASPRFPPPVYHASGHVFGRSHAGLGGGFDGSHLGIMGRGGYPGSNVGVYSPTPSQITKKMADSKATTAKLEDDAAKELHMFVWSSSASPVSEAGLHVFGNQDFSQDEASRNNDFDAKEVRLLVLPAPDSAAPAPAAAPQTSSSTPTPITSTAAASAPPPAPGAVGTAPEQRGGTPELTRQTYEEYVREDFSFGNSKGLGKDESEKDGPSLSKKFGSSSTTELTPKLVDDRRNQSMPPSRVMIRLILDMVFRKLVRNPNTYSSLFGIIWALISFKWHVKMPKLIDNSISILSDAGLGMAMFSLGLFMALQGRLIVCGYSVAAMSMAIRFVTGPAAMAAASVAVGLKGSLLHASIVQAALPQGIVPFVFAKEYNLHPDVLSTSVIFGMIISLPITLVYYVLLGL

>Mapoly0027s0111.1

MLTALDFWHLVEHIIPVYVLVIVGYVLTRFEIIEPVHSPGISKWVSTLGVPMYVFHLLAFNDPYQTSMRLVGADVLSKALVLIGGCLWWRFCKCGSIEGVIGFFMLATLPNTVLIGDALLVPLYGEGAYTQVVTIIFLQSLVWYNLCICLYELRVVLLEEKQNQLMSKSTLELEITRVHHSAFVTVGGSGPLDAVGPAKSDGFDPRHKKRKLHSAFVPPGNQDPTLERTISAPARFSFSRSLSLRFTPGIRVFDLDYDPQNNDVVHPDDKGTPAPASPVMSALDQQNEKTARNVFEQQTEKSEPDLENGNSDFEAEHAEKKTVVLKRSHLSRWALITFKMINRVKKVPLTYASVAGLIYSLFAFKYNWNMPYPIRKSVELISEGALGLAIFLMGMAWARSGRLISCGFKALSYGIVVRFLVGPIIMIIAGFALRLQGTSLKFAILQAVIPQGVISFVLAKEYGLDVPLFNTAVVFQLVIFIPIVLAWYYLMEVF

>Mapoly0027s0108.1

MLTWSELWLLIQHIMPVYVIVLGSYVLNKWDIILPEHGPGLKSWVSTLGVPLYTFHLLAFSDPYQISFQIIGADVIQKVLALLLSGLWWKFSKSANIDGFIAFFMLATLPNTVLVGEAFLKPLYGEEIQGSLVTIIFLQSLIWYNICTVFYELRVVLQDLKEDSGQPQDVPKPELVLSSRRNPTSIPTNGEVLDTNHLPMDEDCEDLESDILERFRSSGQGVDDHLSRNSQNQDPSTHEIQQTLSSPGKCYLLDRGVDLHADAVERDDVGSQNAKIRFIIHKMLNRLKRVPLTYASILGFTYSMLAFKYHWEMPYPVRTSVELISQGCVGMALFLLGMQWARSGQDLLPGGWRALLYGMLVRFAVSPALMLIASLSVGLSANRPDLRFAVLQAVLPQGVVSFMLAKEYGVGVNLFSTAVVIQLLIFVPIAISYYYVLEQI

>Mapoly0053s0108.1

MTGEDFWLLLQYILPFFIILLPAYGMARTKILTQDHALGLNRWTSLVGVPIYVGHMVATNDPYQTNLRLVGADALQKIVVLLIALVWWRFSKRATIDGVITFFMLSTLSNMVLVGPALFRPLHGTASDVSIATIILLQCVLWYNLCIAMFEIRNVMNEMGGKDNETLKNESANTEEPHPSALPGLPLWSSTESSVLPTNKDARSLKRSISAPTRVSFSTSRIVPLFNFDQVEEDSSSKDGTINKPKDIQITFDIPGGVVTKTIENTQIIQIETPETAAQRQPTRAELSRFTYVKIVAFKVVNRVKYAPLLYTSSIGFIYSLFAFKYNWTMPYTIITPIEMIAKGAIAISVVTMALTWSQSGKLVSCGYKRMFYGWFIRFIVGPVIMVITSLAFGLKGISFEFSVLQAALPVGLVSFVLAKEYKLDVEVFSTAVITQLVIFLPIVVVYFFILQVVE

>Mapoly0027s0112.1

MGLKESEVMNLVNAVVPLYISIGLGYTLSKLKVLSYEVHYQGIVTFCFKVTTPPLVFQMVANNNPYLLNLRLIGADVLCKAIVLVVLGLSCVWRPTAQHVAWVSVYFNLATMSNTVLIGIPLLTALYPGTEKDITALVFLQCLIWFSVCIFILELHKVLVQYDPKQAHEDPVHAVVDVEKEGGEPGFGGNDLMNVEHRIGARVAQGSPAAGDESALKHTLAIDLQTKSVTPIPEARVLRVSKFYLVKRITMTVAMNFVKSPIVWSSIIGFTYALLNFRFNDGKPLPVIIKSSVALLANCTLGMAMFMLGMHMAAQPTLISCGLHRALMGAVLRFFVSPAVMAVASTIVGLHGEIFRFAVMQAMIPQAITSFVFACNYHVHEDIFSTAVWFQTLIFFPFALALYTILNLSA

>Pp3c23_10200V3.1

MINGHDIYNVLSAMVPLYVAMMLAYGSVKWWGILTPQQCGGINRFVSIFAVPLLSFQFISGNNPYAMNFKFIAADAVSKVLVLLCLGLWARYAKRGSLEWMITLFVLITIPNTLVMGTPLLAAMYGAGPGDLTVQAVVLQCIIWYTLLLVMYEYRAAKILIMQQFPENAASIVSFKVDSDVMSLDGREPVLTEAEIGDDGKLHVKVRRSVSSRSQGMHSAHHSMPSSKALTPRPSNLTGAEIYSMHSSVNLTPRDSSFNQGEFHSMMSQRSPHRQSNFDTSDVYSLQSSRGPTPRSSNFNEENSKDIHTHHRGLNMNSPRFAPPLYRNGMGARMFTPRPGLGGIGVPGTDCTGHGTLSTLGAPGMGPDGRTIYPGSQTAINILTLGGAANVNATAPSTAVNTQIVNPVYSPQASQIAKKVKDPKASPRADEDAKELHMFVWSANASPVSEAGLHVFGGNDTSANLQQRFDPKEVRMLVHPQLDRGLAAASPRTYDEYTREDFSFGNRNDLKLEDLDKDGPRLDKFGSTSTAELTPKLAEDEAKKSMPPSAVMIKLIAVMTFRKLVWNPNTYSSLLGVIWSLVANRWHLSMPLILYKSVHILSDAGLGMAMFSLGLFMGLGDRIIVCGTKMAVFGMALRFLAGPAVFAAASYLVGLRGVPLKVSIVQAALPQGIVPFVFAKEYGVHPEILSTAVIFGMLIALPITMVYYILLGL

>Pp3c24_2970V3.1

MINGHDIYNVLSAMVPLYVAMMLAYGSVKWWGILTPQQCGGINRFVSIFAVPLLSFQFISGNNPYAMNFRFIAADAVSKVFVLLCLGLWARYSKRGSLEWMITLFVLITIPNTLVMGTPLLAAMYGPGPGDLTIQAVVLQCIIWYTLLLLMYEYRAAKILIMQQFPENAGSIVSFKVDSDVMSLDGREPVLTEAEIGDDGKLHVKVRRSVSSRSQGMHSAHHSMPSSKALTPRPSNLTGAEIYSMHSSVNLTPRDSSFNQGEYFSMMAQRSPHRQSNFDISDVYSLQSSRGPTPRTSNFNEENSKDMHTHHRGLNLTSPRFVPPLYRNVAGGRMFMPRTGLGGLPVHGNDPTGHGSLSTLGTPGMGPDGRTIYPGSQTAISLVTPGGTGNIATPLSSSLNTQIVNPVYSPRASQIAKKVKDTRTSPKSDEDAKELHMFVWSANASPVSEAGLHVFGGNDTSANLHQSFDPKEVRMLVHPQSDLRHPEANPRTYDNYAQEDFSFGNRNDLKLEDLDKDGPRLDNKFGSTSTAELTPKVPEDEAKKSMPPSAVMIKLIAVMTFRKLVWNPNTYSSLLGVIWSLVANRWHFTMPLILYKSVHILSDAGLGMAMFSLGLFMGLGDRIVVCGRKMAIFGMSLRFLAGPAVFAAASYLVGLRGVPLKVSIVQAALPQGIVPFVFAKEYGVHPEMLSTAVIFGMLIALPITMVYYILLGL

>Pp3c10_24880V3.1

MINGHDIYNVLSAMVPLYVAMMLAYGSVKWWGILTPQQCGGINRFVSIFAVPLLSFQFISGNNPYAMNFRFIAADAVSKVFVLLCLGLWARYSKRGSLEWMITLFVLITIPNTLVMGTPLLAAMYGPGPGDLTIQAVVLQCIIWYTLLLLMYEYRAAKILIMQQFPENAGSIVSFKVDSDVMSLDGREPVLTEAEIGDDGKLHVKVRRSVSSRSQGMHSAHHSMPSSKALTPRPSNLTGAEIYSMHSSVNLTPRDSSFNQGEYFSMMAQRSPHRQSNFDISDVYSLQSSRGPTPRTSNFNEENSKDMHTHHRGLNLTSPRFVPPLYRNVAGGRMFMPRTGLGGLPVHGNDPTGHGSLSTLGTPGMGPDGRTIYPGSQTAISLVTPGGTGNIATPLSSSLNTQIVNPVYSPRASQIAKKVKDTRTSPKSDEDAKELHMFVWSANASPVSEAGLHVFGGNDTSANLHQSFDPKEVRMLVHPQSDLRHPEANPRTYDNYAQEDFSFGNRNDLKLEDLDKDGPRLDNKFGSTSTAELTPKVPEDEAKKSMPPSAVMIKLIAVMTFRKLVWNPNTYSSLLGVIWSLVANRWHFTMPLILYKSVHILSDAGLGMAMFSLGLFMGLGDRIVVCGRKMAIFGMSLRFLAGPAVFAAASYLVGLRGVPLKVSIVQAALPQGIVPFVFAKEYGVHPEMLSTAVIFGMLIALPITMVYYILLGL

>Pp3c14_8850V3.1

MLTGGQFYDVLCAVVPLYVALFLGYGSLKWWGVVTPEQSAGISRFNALIAMPPLIFEIIAFNNPYTMNNRLIAAYCLSNGIVLVGLCAWVWCTKCGNLDWVITLFQLSVMPNTIIVGIPVLSPLYSVTESGIAAIFIGQVLWLFPTLFLYELKEVRKMGQPAVGSVAQVQDDGPRSLHSGETTESAAEHGFEGHESGIPALLTQGEHNDFRIGIGDEPSHAATEVSHKDEEHVKNGSRQRNSFSVANGESGTRENGTEHGHEMAPSQMNLKEMAIKVAKKMVQLPLTHATVMGIVYSLIAGRWGFDPLRILRNSLDIMGRITLGLTMYSIGLFMAGQKKLVASWWVAFYGAFCRFIVGPGTMGVASLLLGLRGDTLRFAFLQAALPQAVISFVFAKDYNLHTDVFTTAVSLQTIVFMPIVLVYYTLLEL

>SmPIN99301

MISLHDLYTVLSAVVPLYVAMILAYGSVRWWGILTPEQCGGINRFVAIFAVPLLSFEIVSSNNPYAMNLKFIAADIVQKLAILAALALWVKLSRHGSLEWLITLFMLATLPNTLVMGIPLLAAMYGAKPGGLIVQAVVLQCVIWYTFLLFLYEYRSAKLLIQEQFPDTAGSIISFKIESDVVSLDGREPLQTDAEVGDDGKIHVTVRKSVSSHHSMLSPRRSVGLRSMPSSKALTPRPSNLTGAEIYSMHSSRNLTPRESSFNRNDFYSLMSFTNIPPPRQSNFTNTDVYSLHSSIFPKPGDGGFGFSPKHHHGGELRASPKLVHNNRSAVAAEDPNAKDLHMFVWSSSASPVSEAHPQHQQQQHGGVLGGYVPGRQYDEYDRDDFSFGNRPSFRGDESMIADKEGPSLTKIGSSSTAELAPKDGDNKAEMPPARIMTRVILSMVWRKLVRNPNTYSSLIGVIWALISFKWHIEMPVIVDKSITILSRAGLGMAMFSLAGLFMALQKRLLACGTSLAIYGMVVRFLTGPAVMAAASIACGLRSVELKVSIVQAALPQGIVPFVFAKEYNVHPDILSTAVIFGMLVSFPITLLYYVLLGL

>SmPIN102666

MISGGDFYQVMAAVVPLYVAMILAYGSVRWWGILTPEQCSGINRFVALFAVPLLSFQIISKNNPYLMNPQFIAADAIQKAAVMLVLAIWARYSSRASFEWVITHFMVATLPNTLVMGIPLLYAMYGEKHGSLVVQAVVLQCIVWYTLLLVMYEYRSAKILILEQFPDTAASIVSFKVDSDVISLDGRDQPVLTEAEVGDDGRIHVKVRKSTSSAQSARSPMASMFSSKAGTPRPSNLTGAEIYSLHSSRNMTPRDSSFTRGDAYLMSGGGNSSLMSSNIFGRASTGHHAGGTYSPSPSMQLPKKVANNNISSTTGKDNSATNVLQGIPQGKSADYDAKELHMFVWSSSASPTSEKGLHVFGGTDFNARDGAKFDPKETKLYLHQEFSFADRNGAYDPSDKLEPNLAKLESSSTVELAPKTPNLNGGGTGEGNQAMPPATVMTRLILDMVWRKLVRNPNTYSSLIGLVWALVSFRWNVKAPKLIEKSITILSDAGLGMAMFSLGLFMALQPRILACGTSMAMFGMLLRFFCSPAIMSVASIAVGLRKLDLRAAIVQAALPQGIVPFVFAKEYNVHPDVLSTAVIFGMLVALPITLLYYVLLGI

>SmPIN119024

MITLQEFYTVMSAVVPLYVAMILAYGSVRWWKLLTPAQCSGINRFVAIFAVPLLSFQIIAHNNPYDMNPRFVAADVLQKLIVLAVLASWSRLKKFSSSVRSTSDTKSKDLDWAITLFMVSTLPNTLVLGIPLEVAMYGEKPAELVVQAVVLQCIVWYTLLLFLYEYRSAKNLILEQFPGPSAANIVSFRIDPDVISLDGEQQQIKTEAEIGQDGKIHVQVKRSPPPQPPPLPPTSSMYREYDSSSISLLRSPGLVSPLSSKAGTPRASDLTGVEIYSVQSSRNMTPTGSYTDLGLMTGTHGSSPHLRPSSNNPGLSIETSTTLSSNELPHYSNHSSNMHTPREPPSEEQTEAARKMDYQQQQYESREMQMLVWSSSNSPASDRGCYETRKLTSHKNLSIQSKYVSISVSRKTYILTYEEDTTPLSEKNLKSESHLPCKVPFTPPKLVDSDSSAPTQMPPPTVMTKLIVRMMWKKLIRNPNTYASLLGLAWALISFRWNVGMPKILEHSITILSDAGLGMAMFSLGLFMALQSSLIACGTTMAVVVMIIRFVTGPAIMSATSIAVGLRNVQLRASIIQAALPQGIVPFVFAKEYNVHPDVLSTAVIFGMLISLPINLLYYVLLGL

>SmPIN231064

MISPAEFYSVMAAMVPLYVAMLLAYASVRWWGILTPDQCSGINRFVAIFAVPLLSFQIIAHNNPYDMSLKFVLADALQKIIVLAVLGAWARYSRRGSLEWMITLFMLATLPNTLVMGIPLLNAMYGGDPSRLVVQAVVLQCIVWYTLLLVLFEYRSAKILVAQKFPKPAAAIASVKADPDVVSLSFKEGLTTEASVGEDGKIYVQLKRSPPPIRTAPATKNPFFPPAAMASTAFSSKASTPGASDLSNAEIYSVHSSSVLTPGHSFHRSSDRRLLADGGADHQEGQRRGGRVLQLGLRCERAPDVRVECEYVAEVASVGGCSGEALMAEGRETERNRPWIKTFLPSKDAAAKLPDDPDMPSTAVMTKLILDMVWRKLVRNPNTYSSLLGLTWALISFRWNLRMPKIIEGSITILSDAGLGMAMFSLGLFMALQPKLLACGTSMTIIGMVIRFVTSPAIMSATSIAAGLRNMDLRASIAALPQGIVPFVFAKEYNVHPDVLSTAVIFGMLVSLPITLLYYVLLGL

>SmPIN268490

MISPAEFYTVMAAMVPLYVAMLLAYASVRWWGILTPDQCSGINRFVAIFAVPLLSFQIIAHNNPYDMSLKFVLADALQKIIVLAVLGAWTRYSRRGSLEWMITLFMLATLPNTLVMGIPLLNAMYGGDPSRLVVQAVVLQCIVWYTLLLVLFEYRSAKILVAQKFPKPAAAIASVKADPDVVSLSFKEGLTTEASVGEDGKIYVQLKRSPPPIRTAPATKNPFFPPAAMTSTAFSSKASTPGASDLSNAEIYSVHSSSVLTPGHSFHRSSDRSDFYLMTPSSRSGTIISSRWTCTELEGFSQTAVQITKKDNNEEGASSNLDYDAKELQMFVWSASTSPRSPASVAAPAKLLWRKDEKVLMSHETTLEVPEEAVTRPDGSAAAAVQTPAERNRPWIKTFLPSKDAAAKLPDDPDMPSTAVMTKLILDMVWRKLVRNPNTYSSLLGLTWALISFRWNLRMPKIIEGSITILSDAGLGMAMFSLGLFMALQPKLLACGTSMTIIGMVIRFVTSPAIMSATSIAAGLRNMDLRASIVQAALPQGIVPFVFAKEYNVHPDVLSTAVIFGMLVSLPITLLYYVLLGL

>SmPIN234325

MISAHDFYVVMSAVVPLYVAMILAYGSVRWWKILTPDQCGGINRFVAIFAVPLLSFQILATNNPYKMNAQFIAADVLQKLAILAVLLVWCRYSSNGSMEWTITLFMLATLPNTLVMGIPLLAAMYGTESGSLVVQAVVLQCIIWYTLLLVFYEYRSARNLIRDQFPKTAASIVSFKVDSDVVSLDGNREPIQADAEIGDDGKIHVTVRRSTASRHDLYPPSPIASSKALTPRPSNLTGADIYSIHSSRNMTPRESSFNHAEYYSMMMMMPAFNNPPPPPPPPPPRHSSFTASDIYSLHSSRGPTPRTSNFNIEEQHRPSTKSAPPPSRMIPPPVPAPPPAPAPLAPAPVLNAAASKNDLVHMFVWSSSGSPISEGNVRMLNDDFRSNNNKDVRLLVGDDDDSTATAASGAPHDFSFGHAGGVDEGNSSTNIPARNSSSGLEMPPKNSSNLADDQPTEMPSVGVMTRLIVDMVWRKLIRNPNTYSSLLGLGWALVAYKWHVRMPAIIDNSIQILSKAGLGMAMFSLGLFMALQKRILVCGTSMALFGMVVRFVTGPAVMAATSIAVGLKGVNLHVSIVQASLPQGIVPFVFAKEYNVHPDVLSTAVIFGMLVAFPITMLYYVLLGL

>ScPIN005827

MITGGDMYTVLSAMVPLYVAMILGYASVRWWRIFSAVQCSGINRFVAVFAVPLLSFTFIAGNNPYKMDGAFITADTASKVAALVALVAWAMARRLWGGGTCVDSGGARSRGTGWLWRQSAGKFGGGSGLDWLITLFSLATLPNTLVMGIPLLRAMYGDAYGNLMVQVVVLQSIIWYTLLLFLFELRAAMVLLKERLAAEMEGGGGAAGTAAEIVGVDVDPDVVSLDGREPLHTEAHVGEDGKIRVLIRRSVSVSSSSRFHYHHQLLHHHHGHDLLSSGAAASSSPPRRSAAANFYSPHRPSASAAPIADFYVPSSPGRRSTSVMNLDLYGASPRSVAMMDMAAGPGGAVYGASNNSPRRSSATPAMAQVDNNPAEQQSYYSSGSAAMPIARDLTLGFTSTFTEPPHLRSSISSLPYHHLSPRNSNFASANYDLNYAVNPNTGLNYSRQSSHCPTPRTSMTSIPPTAGLANNHAVANYALNPAAAAALAAEHNSQYVARRDRDLPLEAVESNQLMNTKLQADPKASQSIESLMGDMSKIKTNRNVNHGCEPQSIENILGNDDNVDQISSSNANHMQNNTSGKLQRCGSTNNNNNNEKSSTHIEDVLTTRAVLPSTSIMAKLILKMVSRKLIRNPNTYASLIGVIWSLIAFRYGIGMPKIISQSLQILSQAGLGMAMFSLGLFMALQPKIIACGTYVAIFAMVVRFLIGPMIMSVTSIIVGLRGVHLHAAIVQACSLLNFLSLFVIIVLRKDDCRPLAALPQGIVPFVFSREYDLHPDILSTAVIFGMLVALPTTLLYYVLLGI

>ScPIN011672

MITGANLWTVVTAMVPLYVAMGLGYGSVRWWSVFNADQCAGINRFVAIFAVPLLSLWFIHGNNPYKMDREFLLADAASKGVALAALAAWWWRRRRLDWVITSFSLSTLPNTLVMGIPLLDAMYGPHYSSLMVQIVVLQSLIWYTFLLFLFELRAATSRPSMRLPMPTVMPPIPLDNMNPDDLQVQVSSTCKNGSVDLEVVVSQEEPTNLNHVIAEVEPSEDQVELNHAIAEVGVATEHGNERRLKAMNVLKVVVWKLARNPNTYATLVGIVWSLIAFKYNIGMPKIVQGSIQILSQAGLGMAMFSLGLFMALQPKLIPCGTRLCILAMVMRFLVGPMLMAATSVAIGLHGVQLRAAIVQAALPQGIVPFVFAREYNLHPDILSTAVVFGMLVALPIMLLYYFILEI

>CbPINa

MEVKNLVNVVNAVIPLYFAILLGYLSVKPWNIFTQEQTKSLNTLNAVFVIPAFSFQFLAKTAIYDMNLIFVLADTVAKFGMLVGLGLWVQYLTAIPTVSGKWEWFVVSFMVASVANTLIIGIPLLHAMYGSDSATLLAQVVVVQLVLWFPVLQILSQLNTSIRSTEALKDLEDHSVAGSITSSGVADASVVGGTAPCHQDTAVQVQVEKGSNLRGDAVEQQQLRQKLAKENFKCIGGEGADPPNSFERVEERGGKVPKGETDGKRGPGVASITMQIASIRKKFGPWVNKLRSAALHHHFSGGKSTSKDSVSDYLKKKKTSSSSFITKQCKHTQPIQAVSPNGRPSPAAADTSARSPRADLRQMVMAPGACDTSGLAANSSGGLRGVGQGSTAFNPGLRESCHPLETPQHGELSPAVGESAVKLLNEESSTSDSGQGSPGMAAPAQLPAASVAGPLESQSRSESGLDCRFNACQLAQSVDTDTEAAGISDRAWCSAGATFDGCDPAATGPTLSRLGGEVPDCNLAGPPPSDSLDHQSSTDGTKCIQLDMSSTGVPTAHAGMHIITIKPPTGPLDSAPGASAPATTSLEGSMSREAKSRGQAKHARSCNGEEKPSNLPQLASRRKSEEKVEMAENGGGAAASAEDRGRLSATPGGAEGGKSDQHQQQITGQAGSRSAPLDFSSRLMPEFQTSSSGAGSGGGGGGGGGGGQVLTGDKEDKDFVTKSANYDNYPHSTYRQTLVPSLAISFMPARGDESGPVIEHPKSLWSQPGLATTVTPQQLHAKSQLIAEREERVNTVPVDAIVITSARPTAELLTPILSPTPTPIPVPVQVTGALRNMPIGERGRKKGRRPSVRMSRAEIKQAVKASANSFLHSQLVWASILGLTYSLIAGKLGFKMPTIIDKSLTLMANLCLGLSMFSLGLFMALQSKLIPIGAWTTTWTFSLRLFWGPLVVTIVTYLFGCRGLLLKVAFIQNALPQAVLTFVLANQFHLRADVLSTGVCFGTLVSLPLLIIYYVILEKIL

>CbPINb

MNNLNAYFALPAYSFMVIATCDLTKMDKVFIIADTIGKIVLLISLGFCVRSLPKKHTFASRFQWWATSFMIATITNSVIIGFPLAEAMYGADAIPLMAQLVVVKGVLWFPLVHMLCQMCPIFWKREDHNKLDPPGGTKVKPSCLSSSQSPVTAVSEEILNMPRPSDHGGKSNSELASIAVVRQSVHRRRDANGADNPHAIDPYQWKGDSDPMHKVEVVTGSPSREEELCLKVEMYDATSVTGPVRAAPSVSADATSLREFGQANKATAEVQRTSSDTADKLKLSCSSVSLEAATTAEMQIQVQMPVMGQTHTVTRQNPGSADEEEAAQVGETSRAAGWRCVPAGSDETTEAASRDPQGTNACGLLQSRMNFPMSLSAHRSARKMDPKMPLGEEGSTQCQPHASGKLNPKLESEKDPTSDSEVCKVVRLTAKAFLKQRLLFSGLLGVAFSLLASKYGFEMPTVMRKTVSMLSNMCLGLSMVSLGLFTGCQASLIPIGWWTTTWTFSLRFVWGPLAVAVISYLFGCRGLTLKVGLIHNALPQAVMSFVLASQFDVHPDVLGTGVSFGTLVSMPLLIIYYVVLELLL

>CbPINc

MLATVPNTVIVGFPLLEAMYGPDSLPLVAQIVVVQVVLWFPFLQIALDIAQSLRKPERRPTEDSESNFATLQSRQSPSIFSLAARGDRPDREMLQRSTATDPCCSNHRHVAVARIITEASAHDSRESAVSGAEGNHDYGSANRDDDREREEGRSLETLPIRSTSSFFTESPADSAEAGSSHLDGLEPSISVVIQASGGSAPPRSPPAAAALRRLTARASGQESESPLRDQCREQTQKRPGVAGDGVQDVIEETKGWRCVPAGSNCCEMEAGANTKDGRRPCPWLPSSSPHQSDQSASRLPSGPSQSSNPDGADGPDPLEGGNEEGPASPGPSGPSQSSNPDPRSPRSSGERRLWVVCVKAFLQGRLMWAGLVGMAYSLLASRYGFDMPTPLEKTVTMLSNMCLGLSMISLGMFTALEGSLVPIGWWNTTWTFSLRLLWEPALAGIITYLFGCRGRVLKVAIIQSALPQAVITFVLASQFGVHPDVLSTSVGLGTLVSLPLLIVYYLLLELLV

>PSC76620.1 Micractinium conductrix

MGLGLKTDLYSEQIWRFVGAFLMMRGIMLAACAVFFGLVLRCSLGEVTANWLSTTWISTVILGTPLLRALLGPKYANLGVVAGISSFIFQLPVMLILFEVHTWRQETLAAALPQTQSGDKALASPPSPPIDGTEQEVVASKPGDESLPGSSAPGAIGAQRVHPEGLGSATVPPPAGGLHSTGSWLHFKMTRKQGQRLGLRLVMNHVLWGVGIGIILSLSKIGPKWLDPGTPPAQPNCEYAVGAGFIFLLLEYFARCTEPVAFFGTGMWMVRPRPLAIGWWRAALYMLVKLVLVPMLMVGCCFAVGLEGAYARAAVLVGTLPVSAAAFALSKTYNVGEDVAVANVFLAHLLVLPTTIAWLEAMDAMGLFTASFGKLPPTCTNPCLAGPPAGVPLPALCKNMELQQVDATSMRTHLDQAVDAAVDRLLAQNASLPHGELLAEARQAKAEAEGLRQALAVDDEEFKRLYAVVAELEAKCAELQRQQQQQHPAQQQQQPAQQQQQQQQRQQQQPAQQQQQQQQQQQQQHAVEAEPSPREQQLEEKLAKVKQQLESAGAKLVERDIELAAAKRAQAEAVAALHQAESDRRRVQVQLERQAEELAGVQREAALLRARKTAVDAAARIAASAGAAPAAAQQAQQQAAAQAAQAAPPAPPEPAAAATAAALPPLLQELKQQIEASLRQELQQQLGQLSTAAAQQHSHGAPGHTRHSLSPSVAQPPAPAGALHAQVGSPQGPPQQQQAQQQAGAGSLGGQSRAPSPPSSGEVEMEQQAEQQQQQQQRQSPQQRQQQHTEQQQQRQSPQQWQQRQQHAEQQQQQQQQQQQQQRQQRQQQQQQQQQQQRQQRQQQQQQQQQQQQQQQQQQQQQQQQQQQQQQQQQQQQQQQQPPISFSNPAVAAAIAAAQALSAELAAKQGAGKAAAGQACVQAATGQGQATAADDELPPWLALAAGTRRQQAAATAAAAAQQQQQQQQLDLQAAAHRREQVARWRELARSYNAAHGGAVSRPLLSLPFAVQPDADAEEIAHRTGVGELPYACPPCREVFDTELEYLEHQSTRQHSDTCRTLYRAWTGNSLDAQGIARNSKSPPKVQLKSHQRAKKFCCLACSRTFQMEQFVQHLACSSHVSCAQRQAAAVKAAAGGGKRQKTGGG

>PSC76619.1 Micractinium conductrix

MWKFQEFLNADIAIMLVIIAGAASVWRNLIFKREDMRVFNLFLFKATLPASVIMGLGLKTDLYSEQIWRFVGAFLMMRGIMLAACAVFFGLVLRCSLGEVTANWLSTTWISTVILGTPLLRALLGPKYANLGVVAGISSFIFQLPVMLILFEVHTWRQETLAAALPQTQSGDKALASPPSPPIDGTEQEVVASKPGDESLPGSSAPGAIGAQRVHPEGLGSATVPPPAGGLHSTGSWLHFKMTRKQGQRLGLRLVMNHVLWGVGIGIILSLSKIGPKWLDPGTPPAQPNCEYAVGAGFIFLLLEYFARCTEPVAFFGTGMWMVRPRPLAIGWWRAALYMLVKLVLVPMLMVGCCFAVGLEGAYARAAVLVGTLPVSAAAFALSKTYNVGEDVAVANVFLAHLLVLPTTIAWLEAMDAMGLFTASFGKLPPTCTNPCLAGPPAGVPLPALCKNMELQQVDATSMRTHLDQAVDAAVDRLLAQNASLPHGELLAEARQAKAEAEGLRQALAVDDEEFKRLYAVVAELEAKCAELQRQQQQQHPAQQQQQPAQQQQQQQQRQQQQPAQQQQQQQQQQQQQHAVEAEPSPREQQLEEKLAKVKQQLESAGAKLVERDIELAAAKRAQAEAVAALHQAESDRRRVQVQLERQAEELAGVQREAALLRARKTAVDAAARIAASAGAAPAAAQQAQQQAAAQAAQAAPPAPPEPAAAATAAALPPLLQELKQQIEASLRQELQQQLGQLSTAAAQQHSHGAPGHTRHSLSPSVAQPPAPAGALHAQVGSPQGPPQQQQAQQQAGAGSLGGQSRAPSPPSSGEVEMEQQAEQQQQQQQRQSPQQRQQQHTEQQQQRQSPQQWQQRQQHAEQQQQQQQQQQQQQRQQRQQQQQQQQQQQRQQRQQQQQQQQQQQQQQQQQQQQQQQQQQQQQQQQQQQQQQQQPPISFSNPAVAAAIAAAQALSAELAAKQGAGKAAAGQACVQAATGQGQATAADDELPPWLALAAGTRRQQAAATAAAAAQQQQQQQQLDLQAAAHRREQVARWRELARSYNAAHGGAVSRPLLSLPFAVQPDADAEEIAHRTGVGELPYACPPCREVFDTELEYLEHQSTRQHSDTCRTLYRAWTGNSLDAQGIARNSKSPPKVQLKSHQRAKKFCCLACSRTFQMEQFVQHLACSSHVSCAQRQAAAVKAAAGGGKRQKTGGG

>XP_002295236.1 Thalassiosira pseudonana (Marine diatom) (Cyclotella nana)

MTWVYLPVLNALIQILITIALGFVSDFFGIVSADRLVPEAVHLVFYVLLPSLIINGIGIQIDLYTESNVWAFIVAFLILRAIALILSLAIALFVNWNQQQRVLGLGDVAVLWLSLSWISTVILGVPICTAVFENPTLGAKYGIMAGISSFIFQLPLQLMFLECHAAEEAQRTSEATGRSTNSAREELPSQEKERESAVITIQEASEESFHQLSTEDIAPTAIGDRIQEAELHLRRSWWSLVYAEHLPDMDLWLDILRRVLKNPVVDGIFVGIVISLSTAGRYLRCPSDTCVEGLEWISATLGWLGNCVSPLSLFAMGAWMHSQRKLILIPIPNLCVAMVSKLIVVPLLMVGLAKGMKLNNESARAAVLIATLPISLASFSLARQYNVGEKDLAANVAFGTLLMLPTVIVWNIVLDSVGLYPIEIV

**SUPPLEMENTARY VIDEOS**

**Video S1.** Time-lapse imaging of *Chlamydomonas reinhardtii* cell division in Tulip microfluidic chambers under mock conditions. Recording starts at 0 h and ends at 10 h. Scale bar = 10 µm.

**Video S2.** Time-lapse imaging of *Chlamydomonas reinhardtii* cells in Tulip microfluidic chambers under 50 µM IAA treatment. Recording starts at 0 h and ends at 10 h. Scale bar = 10 µm.
